# Alba-Domain Proteins of *Trypanosoma brucei* Are Cytoplasmic RNA-Binding Proteins That Interact with the Translation Machinery

**DOI:** 10.1371/journal.pone.0022463

**Published:** 2011-07-21

**Authors:** Jan Mani, Andreas Güttinger, Bernd Schimanski, Manfred Heller, Alvaro Acosta-Serrano, Pascale Pescher, Gerald Späth, Isabel Roditi

**Affiliations:** 1 Institute of Cell Biology, University of Bern, Bern, Switzerland; 2 Department of Clinical Research, University of Bern, Bern, Switzerland; 3 Liverpool School of Tropical Medicine, Liverpool, United Kingdom; 4 Department of Parasitology and Mycology, G5 Virulence Parasitaire, Institut Pasteur, Paris, France; The University of Maryland, United States of America

## Abstract

*Trypanosoma brucei* and related pathogens transcribe most genes as polycistronic arrays that are subsequently processed into monocistronic mRNAs. Expression is frequently regulated post-transcriptionally by *cis*-acting elements in the untranslated regions (UTRs). GPEET and EP procyclins are the major surface proteins of procyclic (insect midgut) forms of *T. brucei.* Three regulatory elements common to the 3′ UTRs of both mRNAs regulate mRNA turnover and translation. The glycerol-responsive element (GRE) is unique to the GPEET 3′ UTR and regulates its expression independently from EP. A synthetic RNA encompassing the GRE showed robust sequence-specific interactions with cytoplasmic proteins in electromobility shift assays. This, combined with column chromatography, led to the identification of 3 Alba-domain proteins. RNAi against Alba3 caused a growth phenotype and reduced the levels of Alba1 and Alba2 proteins, indicative of interactions between family members. Tandem-affinity purification and co-immunoprecipitation verified these interactions and also identified Alba4 in sub-stoichiometric amounts. Alba proteins are cytoplasmic and are recruited to starvation granules together with poly(A) RNA. Concomitant depletion of all four Alba proteins by RNAi specifically reduced translation of a reporter transcript flanked by the GPEET 3′ UTR. Pulldown of tagged Alba proteins confirmed interactions with poly(A) binding proteins, ribosomal protein P0 and, in the case of Alba3, the cap-binding protein eIF4E4. In addition, Alba2 and Alba3 partially cosediment with polyribosomes in sucrose gradients. Alba-domain proteins seem to have exhibited great functional plasticity in the course of evolution. First identified as DNA-binding proteins in *Archaea,* then in association with nuclear RNase MRP/P in yeast and mammalian cells, they were recently described as components of a translationally silent complex containing stage-regulated mRNAs in *Plasmodium*. Our results are also consistent with stage-specific regulation of translation in trypanosomes, but most likely in the context of initiation.

## Introduction

The vital importance of RNA-binding proteins in myriad processes in eukaryotic cells - from RNA synthesis to splicing, export, localisation, translation, and finally, decay - is widely appreciated [1], but there are still major gaps in our knowledge. Interactions between mRNAs and RNA-binding proteins can either be transient, as is the case during processing or export from the nucleus to the cytoplasm, or they can be more stable, such as the association of polyA-binding proteins with the mRNA tail. Gene expression in higher eukaryotes is frequently regulated at the level of transcription initiation at the promoters of individual genes. In contrast, early-branching eukaryotes from the order Kinetoplastida, which includes the “Tritryp” parasites *Trypanosoma brucei, T. cruzi* and *Leishmania*, transcribe their genes as large polycistronic arrays and therefore rely much more heavily on post-transcriptional mechanisms [2], [3]. As a first step, monocistronic mRNAs are generated from polycistronic precursor RNAs by *trans*-splicing of a capped 39 nt spliced leader to the 5′ end and concomitant 3′ polyadenylation of the upstream mRNA [4], [5], [6]. Despite originating from the same precursor RNA, mRNAs from a single transcription unit can vary widely in abundance owing to differences in processing efficiency and mRNA stability [7], [8], [9]. Kinetoplastids have different life cycle stages and many of them, including the Tritryps, cycle between insect and mammalian hosts. In addition to differential control of mRNA turnover, control at the level of translation and protein stability helps orchestrate stage-specific expression during the life cycle [10].

Over the last two decades a number of *cis*-acting elements have been identified in the 3′ untranslated regions (UTRs) of stage-specific or cell-cycle specific mRNAs of kinetoplastids [11]. Recent examples of such elements are SIDER1 and SIDER2 retroposon sequences in developmentally regulated mRNAs in *Leishmania* [12], and a conserved UUGUACC sequence present in a number of transcripts that are coordinately expressed during the *T. brucei* cell cycle [13]. One set of mRNAs in *T. brucei* for which detailed knowledge of regulatory elements has accumulated over the years is that of the procyclins (EP1, EP3 and GPEET) which are the major surface glycoproteins of procyclic forms of the parasite in the midgut of the tsetse fly [14]. GPEET is the predominant coat protein in early procyclic forms, giving way to EP1 and EP3 in late procyclic forms [15], [16]. Stage-specific regulation of procyclins is multilayered, encompassing transcription initiation, elongation, processing, mRNA stability and translation (reviewed in [14]). Transcription initiation is approximately 10-fold higher in procyclic forms than in bloodstream forms in the mammalian host [17], [18]. The 3′ UTR of EP1 procyclin contains three stem-loop structures (LI, LII and LIII), each of which contains a regulatory element. An element in the first forty bases of LI and a conserved 16mer in LIII are positive elements that stabilise the mRNA and enhance translation in both bloodstream and procyclic forms [19], [20], [21]. Deletion of the 16mer reduces association of the mRNA with polysomes [14]. In contrast, a 26mer in LII is a negative element that renders the mRNA more labile and reduces its steady state levels [22]. The 3′ UTR of GPEET shares this structural organisation into three stem-loop domains and contains the 16mer and 26mer as well as an additional element in LII, the glycerol-responsive element (GRE). The GRE has a weak positive effect in early procyclic forms, but destabilises the mRNA in late procyclic forms [23], [24]. In culture, the presence of glycerol to the medium prolongs GPEET expression by impeding the degradation of the mRNA. In addition to elements in the 3′ UTR, procyclin coding regions inhibit expression in bloodstream forms [21] and epimastigote (salivary gland) forms [25]. It is not established, however, if the mRNAs are not translated or, alternatively, if the proteins are synthesised, but degraded too rapidly to be detected.

In general, our knowledge about *trans*-acting factors in trypanosomes is extremely limited and has lagged far behind the identification of regulatory elements in mRNAs (reviewed in [11]). Purification of RNA-binding proteins based on their affinity to a given RNA sequence has proven difficult. Only one component of procyclin mRNPs has been identified to date, the zinc finger protein TbZFP3 which modulates translation of EP1 and which requires both LII and the 16mer in LIII for the interaction [26]. It is not certain, however, if this association is direct or is mediated by other proteins. With the publication of the genome sequences of the Tritryps in 2005 [27], [28], [29], homology-based approaches have offered a way to identify likely candidates based on their functions in other systems. Genes encoding RNA-binding proteins have been identified by searching for known motifs such as the RNA recognition motif (RRM), PUF domains or CCCH zinc fingers [30], [31]. This approach has been fruitful in a few cases, leading, for example, to the discovery that targets of *T. brucei* PUF9 contained the motif UUGUACC [13]. However, the limitation of this approach is that only proteins containing canonical RNA-binding signatures are identified and tested.

The LII domain of GPEET was previously shown to be both necessary and sufficient for stage-specific expression by procyclic forms in culture and in the fly [24]. For this reason we chose it as the starting point to identify possible *trans*-acting factors. Using a gel shift assay as the readout for the interaction, we purified a group of proteins that contain nucleic acid binding motifs known as Alba domains (Pfam: PF01918). These are related to chromatin-associated proteins in *Archaea* and nuclear proteins involved in tRNA processing in yeast and humans [32]. Our analysis, however, reveals major differences in their localisation and function in trypanosomes.

## Results

### Enrichment and identification of proteins interacting with a regulatory element in GPEET mRNA

**Figure 1:**
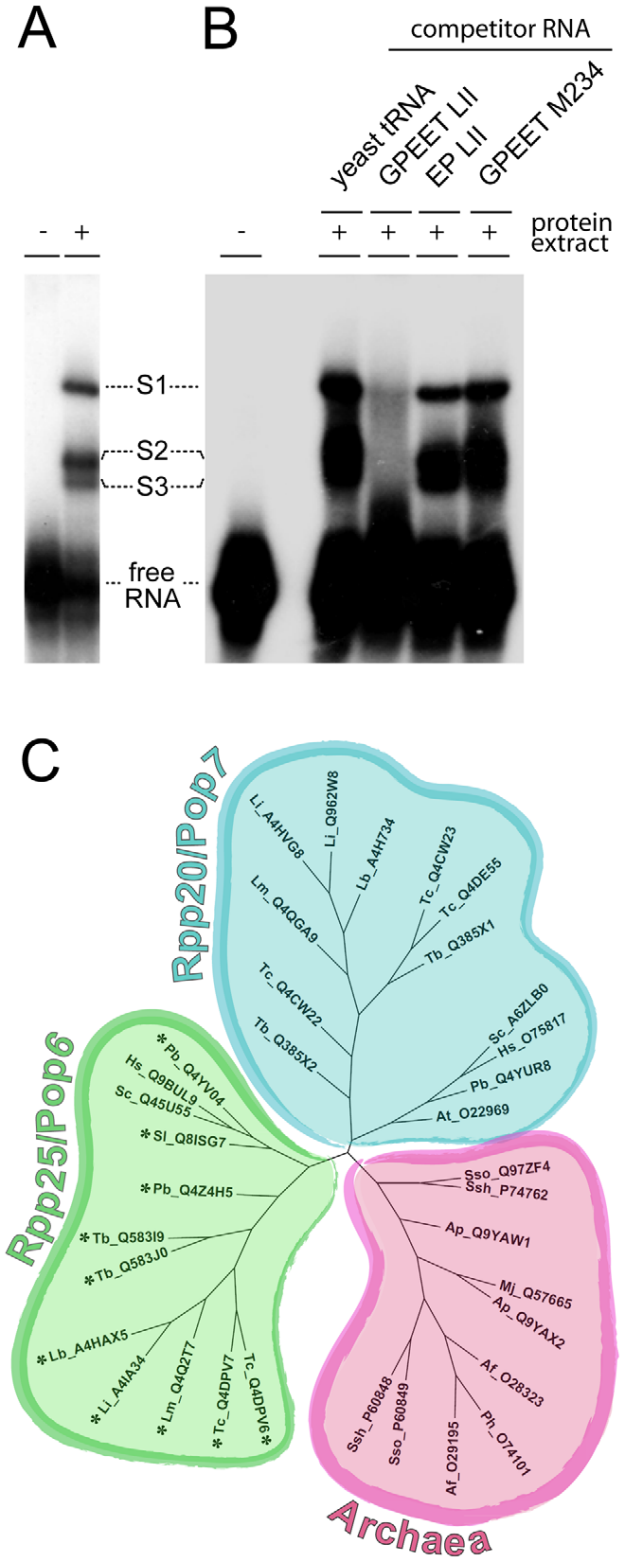
The *cis*-regulatory element GPEETLII specifically interacts with proteins from *T. brucei*. (A) Band shift assay of radiolabeled GPEETLII RNA with protein extracts from procyclic form trypanosomes reveals three shifts: S1, S2 and S3 (B) Competition experiment in which a ∼100-fold excess of unlabeled yeast tRNA, GPEETLII, EPLII or GPEETM234 [24] was added to the binding reaction. (C) Phylogenetic tree classifying *T. brucei* Alba proteins within the Rpp20/Pop7 and Rpp25/Pop6 Alba protein subfamilies. The Alba superfamily of proteins is divided into three major subfamilies [32]. All archaeal proteins group together in one branch (pink). Eukaryotic Alba proteins belong to either one of two branches typified by Rpp20/Pop7 (blue) and Rpp25/Pop6 (green). Phylogenetic analysis based on isolated Alba-domains of a set of Alba proteins places Alba1 and Alba2 in the Rpp20/Pop7 subfamiliy and Alba3 and Alba4 in the Rpp25/Pop6 subfamily. Proteins are indicated with an abbreviation for the species name followed by their UniProt accession number: Af: *Archaeoglobus fulgidus;* Ap: *Aeropyrum pernix;* At: *Arabidopsis thaliana;* Hs: *Homo sapiens;* Lb: *Leishmania braziliensis;* Li: *Leishmania infantum;* Lm: *Leishmania major;* Mj: *Methanocaldococcus jannaschii;* Pb: *Plasmodium berghei;* Ph: *Pyrococcus horikoshii;* Sc: *Saccharomyces cerevisiae;* Sl: *Stylonychia lemnae;* Ssh: *Sulfolobus shibatae;* Sso: *Sulfolobus solfataricus;* Tb: *Trypanosoma brucei;* Tc : *Trypanosoma cruzi.* Particularly: Tb_Q385X1 is Alba1; Tb_Q385X2 is Alba2; Tb_Q583I9 is Alba3; Tb_Q583J0 is Alba4; Sc_A6ZLB0 is Pop7; Hs_O75817 is Rpp20; Sc_Q45U55 is Pop6; Hs_Q9BUL9 is Rpp25 and Sl_Q8ISG7 is Mdp2. Proteins marked with an asterisk contain RGG repeats in their C-termini.

Post-transcriptional regulation of procyclins has been shown to involve several *cis*-acting elements in the 3′ UTRs of EP and GPEET mRNAs [19], [20], [21], [22], [23], [24]. For the present study we focused on the GRE to identify potential *trans*-acting RNA-binding proteins. This element, which comprises 25 nucleotides in the LII region of the GPEET 3′ UTR, was shown to be crucial for GPEET regulation during differentiation of the parasite from the early to the late procyclic form [24]. We established an electromobility shift assay (EMSA) to visualise interactions between the GRE and putative *trans*-acting proteins from crude extracts of procyclic form trypanosomes. For these experiments a radioactively labeled RNA probe (GPEETLII) was generated by *in vitro* transcription. To preserve the secondary structure of the RNA probe, the transcribed sequence included the complete LII region of the GPEET 3′ UTR. Protein extracts from procyclic form trypanosomes were incubated with the labeled probe and separated on native polyacrylamide gels. Autoradiography revealed a distinctive pattern consisting of three complexes designated S1, S2 and S3 (Figure 1A). To test the specificity of the shifts observed with GPEETLII, competition experiments were performed with an excess (∼100-fold) of unlabeled GPEETLII RNA, the corresponding region from the EP 3′ UTR (EPLII) or a mutated version of GPEETLII (GPEETM234). The GRE is absent from the LII region of the EP 3′ UTR and is mutated in GPEETM234. This mutation abolishes regulation when used in reporter gene assays (Figure S1; [24]). Only unlabeled GPEETLII RNA was able to compete with the labeled probe, indicating that proteins from *T. brucei* interact specifically with GPEETLII RNA containing the GRE (Figure 1B).

To identify proteins interacting with GPEETLII RNA, crude extracts were cleared by ultracentrifugation to give an S100 supernatant that was then subjected to sequential column chromatography. We noted that the proteins contributing to all three band shifts were pelleted to some extent in this step, suggesting a partial association with higher molecular weight complexes (data not shown). Sample complexity was reduced approximately 100-150-fold by purification on heparin columns followed by either ion-exchange chromatography or size exclusion chromatography, while retaining high binding activity for GPEETLII RNA. Proteins enriched in the active fractions were analysed by Edman sequencing or LC-MS/MS. Proteins migrating at approximately 16 kDa, 12 kDa and 22 kDa could be identified as Alba1 (Tb11.02.2040), Alba2 (Tb11.02.2030) and Alba3/Alba4 (Tb927.4.2040, Tb927.4.2030) respectively. Due to the high sequence identity between Alba3 and Alba4 and their corresponding peptides, unambiguous identification was not possible. In addition, two known RNA-binding proteins, MRP1 and MRP2 [33], [34] were identified in the active fractions.

### Phylogenetic position of *T. brucei* Alba proteins

The Alba superfamily of proteins is split to three major branches. One branch is the archaeal branch typified by proteins such as *Sulfolobus shibatae* Ssh10b. There are two eukaryote-specific branches exemplified by human and yeast RNase P/MRP subunits Rpp20/Pop7 and Rpp25/Pop6, the latter including the ciliate protein Mdp2 [32], [35]. We performed phylogenetic analysis by analysing amino acid sequences corresponding to the Alba domains of a set of Alba proteins. *T. brucei* encodes four Alba-domain proteins. According to this analysis, *T. brucei* Alba1 and Alba2 group with Rpp20/Pop7 and Alba3 and Alba4 are with Rpp25/Pop6 (Figure 1C). Alba1 and Alba2 contain the FDXh signature close to their C-termini that is characteristic for the Rpp20/Pop7 subfamily. Furthermore, Alba3 and Alba4 both include the short sequence motif GYQXP typical for the Rpp25/Pop6 subfamily and both have RGG repeats in their C-termini, a feature shared with many proteins from this subgroup. The glycine residue that corresponds to amino acid position 43 in the *Sulfolobus shibatae* protein (UniProt accession number P60848) is highly conserved among archaeal and eukaryotic Alba proteins and is shared by all four *T. brucei* Alba proteins (Figure S2).

### Crosstalk between Alba proteins

**Figure 2:**
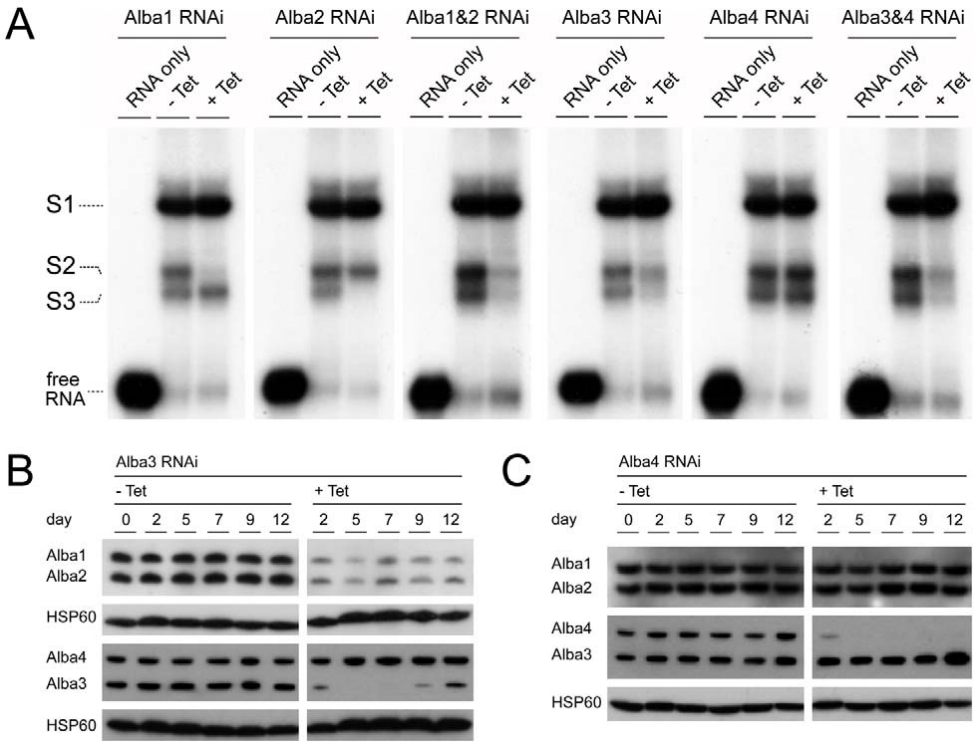
Alba proteins are components of complexes S2 and S3. (A) A panel of RNAi cell lines was constructed to knock down single Alba proteins or combinations of Alba proteins. A derivative of AnTat 90-13 [36], in which one copy of the GPEET coding region was replaced by enhanced GFP, was used as the parental line for Alba 1, Alba2, Alba1&2 and Alba3&4 RNAi cells. Unmodified AnTat 90-13 was used as the parental line for Alba 3 and Alba4 RNAi cells. RNAi was induced by addition of tetracycline to the cultures 3 days prior to the preparation of protein extracts. Band shift assays with ^32^P-labeled GPEET were performed as described in the legend to Figure 1. - Tet: uninduced; + Tet: induced; RNA only: incubation of GPEETLII without protein extract. (B) and (C): Alba1 and Alba2 proteins are dependent on Alba3. Western blot analysis of Alba proteins after knockdown of Alba3 (B) or Alba4 (C) by RNAi. Protein extracts of uninduced (- Tet) and induced (+Tet) cells were prepared every second or third day for 12 days and Alba proteins were detected with specific antibodies. HSP60 served as a loading control.

To verify that the Alba proteins were indeed components of the band shifts we established a series of inducible RNAi cell lines in AnTat90-13 [36] or a derivative in which one copy of the GPEET coding region is replaced by GFP (G. Schumann Burkard, manuscript in preparation). The cultures were incubated for 3 days with tetracycline to induce destruction of target transcripts before preparing extracts for band shift experiments. Knockdown of Alba1 or Alba2 led to a reduction in shifts S2 or S3, respectively (Figure 2A), and an Alba1&2 double RNAi cell line resulted in a loss of both S2 and S3. Alba3-specific RNAi and Alba3&4 double RNAi gave the same result, with a reduction in S2 and S3. In addition, we noted that Alba3 RNAi and Alba3&4 RNAi led to a slow growth phenotype (Figure S3). In contrast to Alba3, Alba4-specific RNAi did not alter binding and had no effect on growth. Since MRP1 and MRP2 were present in the active fractions, we also tested an extract from an MRP1&2 double RNAi cell line [34]. In this case, tetracycline induction caused a loss of S1 (Figure S4). MRP1 and MRP2 localise to the mitochondrion, where they have been reported to function as matchmakers between guide RNAs and to-be-edited target pre-mRNAs. Their RNA-binding mode appears to be largely sequence-independent [37]. Because we cannot exclude the presence of mitochondrial contaminants in the initial extract used for protein purification, and on the basis of the published literature about MRP1 and MRP2, we did not pursue them further for this study.

Studies with *Archaea*, yeast and mammalian cells have shown that Alba proteins occur as homodimers or heterodimers with other Alba-domain proteins and potentially associate with proteins of other families. Several structural analyses of recombinant archaeal Alba proteins point to the formation of higher order structures such as dimer-dimer stacks or extended RecA-like protein filaments [38], [39], [40], [41], [42], [43]. Furthermore, it has been reported that knockdown of either Rpp20 or Rpp25 in HEp-2 cells led to reduced levels of the other protein [44]. Analysis of the Alba3&4 RNAi cell line by immunoblotting showed that not only Alba3 and Alba4, but also Alba1 and Alba2 were reduced after RNAi induction. Alba1 and Alba2 transcripts remained stable, however (data not shown). Analysis of the Alba3 RNAi cell line revealed that Alba1 and Alba2 proteins were co-depleted along with Alba3 after induction of the cells, while Alba4 was unchanged (Figure 2B). RNAi of Alba4 (Figure 2C) demonstrated that this effect was unique to Alba3 despite the high sequence identity between these two proteins. RNAi against Alba1 or Alba2 had no effect on each other or on Alba3 (Figure S5). The co-depletion of proteins after Alba3 RNAi is indicative of physical association between these proteins, which is consistent with the EMSA data. However, an alternative explanation could be that Alba3 regulates the synthesis of Alba1 and Alba2.

### Effect of Alba proteins on GPEET expression

**Figure 3:**
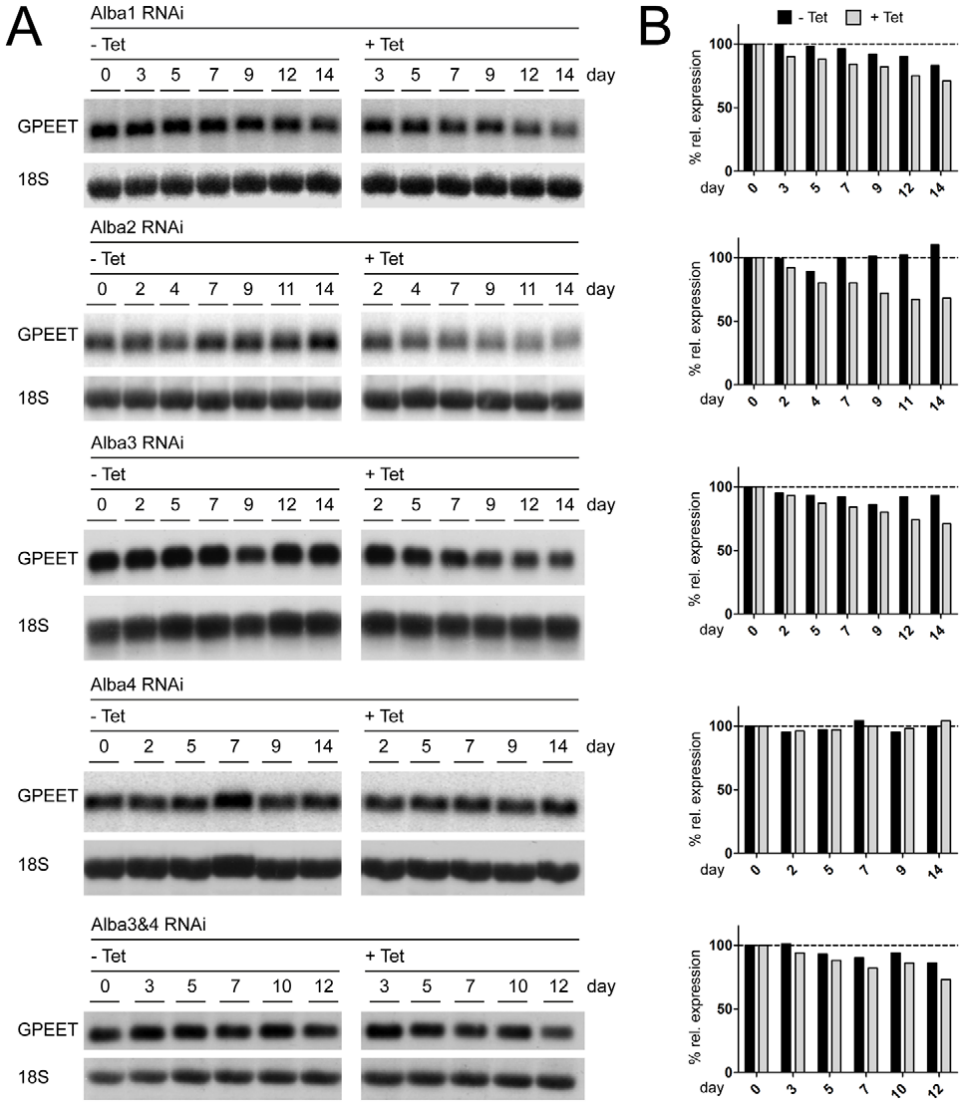
Ablation of Albas has little effect on GPEET mRNA levels. (A) Total RNA from induced (+ Tet) and uninduced (- Tet) RNAi cells was extracted on the days indicated. Blots were hybridised with probes recognizing GPEET mRNA and 18S rRNA, which served as a loading control. (B) Quantification after normalisation to 18S rRNA. Steady-state levels of GPEET mRNA remained constant in the Alba4 and Alba3&4 RNAi cell lines.

Various RNA-binding proteins in trypanosomes and other organisms have been described to bind to *cis*-acting elements in mRNAs and thereby dictate their localisation, stability or translation. We first addressed the question whether knockdown of Alba proteins by RNAi led to changes in the steady state level of GPEET mRNA (Figure 3A and B). Although glycerol was present in the medium throughout these experiments, we observed that the level of GPEET mRNA in the Alba1, Alba3 and Alba3&4 RNAi cells slightly decreased with time, irrespective of whether tetracycline was present or not. GPEET mRNA levels remained steady in the Alba4 RNAi cells during the entire time course and were not affected by tetracycline. Only Alba2 RNAi showed a moderate difference between induced and uninduced cells in the second week of the experiment. Knockdown of the targets was efficient in all cases with proteins being hardly detectable within two days after addition of tetracycline (data not shown). Differentiation from early procyclic forms to late procyclic forms is triggered *in vitro* by transferring the cells to medium without glycerol. In the case of wild type AnTat 1.1, GPEET transcript levels are reduced to approximately 15% after 7 days and to less than 5% after 14 days (Figure S6). This led us to exclude a direct influence of Alba proteins on GPEET mRNA stability.

**Figure 4:**
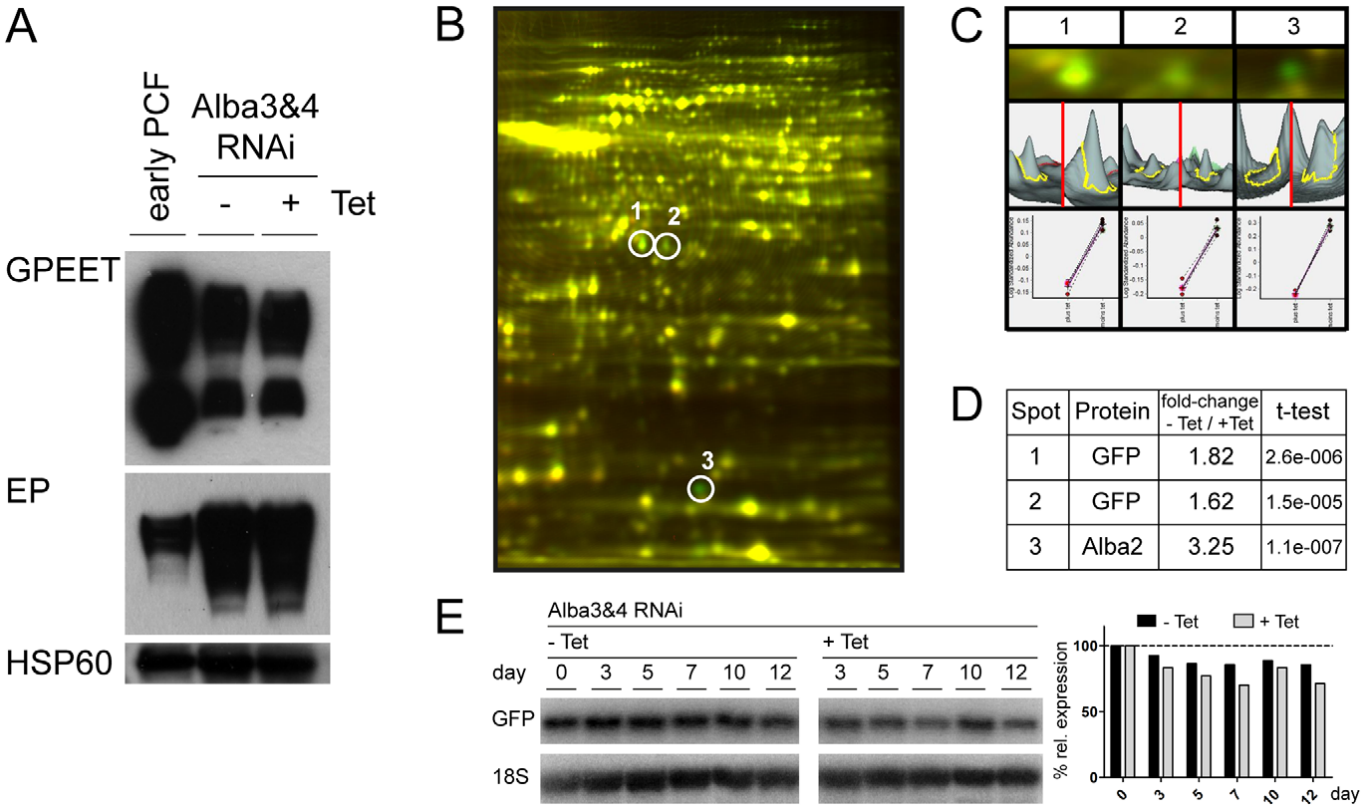
Depletion of Alba proteins does not affect GPEET and EP procyclins, but affects translation of a reporter. (A) Western blot analysis was performed on total lysates (1.5×10^6^ cell equivalents per lane). Alba 3&4 RNAi cultures were incubated for 4 days in the presence or absence of tetracycline (+/− Tet). Western blot analysis was performed with antibodies against GPEET and EP. Mitochondrial HSP60 was used as a loading control. Early procyclic forms of AnTat 1.1 (early PCF) possess two copies of GPEET. In Alba3&4 RNAi the coding region of one copy of GPEET is replaced by GFP and the adjacent copy of EP3 is replaced by a puromycin-resistance cassette. (B–E). 2D-DIGE detects a limited number of differences in following knockdown of Alba proteins. Alba3&4 RNAi cells were cultured for 4 days in the presence (+Tet) or absence (−Tet) of tetracycline, which is before the onset of the slow growth phenotype. (B) Merge of a representative pair of gels (one of 4 biological replicates) showing significantly regulated proteins. (C) Enlarged regions of 2D-DIGE gels for Cy3-labeled proteins from induced (+Tet; green) and Cy5-labeled proteins from uninduced (−Tet, red) cultures, and the corresponding 3D views. The lower panel shows a graphic representation of differences in abundance of these proteins across the 4 independent experiments. (D) Protein identities, fold difference (+Tet/−Tet) and statistical significance. (E) Northern blot analysis and quantification of GFP mRNA in Alba3&4 RNAi cells. The entire ORF of GFP was used as a hybridisation probe.

To investigate whether Alba proteins affected translation of procyclin mRNAs, Western blots were performed with extracts from Alba3&4 RNAi cells (Figure 4A and Figure S7). EP and GPEET were detected, but there were no differences between tetracycline-induced and uninduced cultures. The lower level of GPEET in the Alba3&4 RNAi cells might be due, in part, to the fact that these cells only have one copy of the gene. However, the increased expression of EP compared to early procyclic forms (despite the deletion of one copy of EP3 in this cell line, see next section) indicates that these cells have drifted towards being late procyclic forms despite the presence of glycerol in the medium

To examine whether Alba proteins selectively affected different isoforms of EP, as was previously reported for TbZFP3 [26], we analysed the procyclin repertoire by MALDI-TOF spectrometry. For these experiments the RNAi lines designed to knockdown individual Alba mRNAs were cultured in the presence or absence of tetracycline for 8 days. In all cases EP1 was the predominant isoform and there were no changes when Alba proteins were depleted. For the Alba1, -2 and -3 RNAi cells we observed a decrease in the level of GPEET over time, but this was independent of RNAi (Figure S8, compare day 4 +/−tet or day 8 +/−tet for Alba3). Since GPEET expression can be affected by metabolites, hypoxia or the activity of mitochondrial enzymes [23], [24], [45] these changes might reflect subtle differences in energy metabolism and/or oxygenation when cultures are scaled up for this type of experiment.

### Translational control by Alba proteins is extremely specific

Based on comparative immunoblotting of recombinant Alba proteins and trypanosome extracts, we calculated that there are between 10,000 and 20,000 molecules of each protein present per cell (data not shown). This exceeds the estimated number of GPEET transcripts by more than an order of magnitude [7], [8]. To explore the possibility that Alba proteins are general regulators of translation initiation we performed metabolic labeling of the Alba3&4 RNAi line, in which all Alba proteins are depleted, with ^35^S-methionine. Four days after induction of RNAi we observed a reduction in incorporation of 16% without any obvious changes in protein patterns on 1-dimensional SDS-polyacrylamide gels (data not shown). These cells grew more slowly, however, with their doubling time being approximately 20% longer than that of uninduced cells after 4 days and up to 50% longer after 10 days (Figure S3B). Alba3 RNAi led to a comparable slow growth phenotype (Figure S3A) and methionine incorporation was approximately 10% lower after induction (data not shown).

To improve the resolution of the proteome we performed 2-dimensional differential gel electrophoresis (2D-DIGE; Figure 4B, C and D). Four independent cultures of Alba3&4 RNAi cells, incubated for 4 days in the presence or absence of tetracycline, were used for this analysis. Of a total of 2300 spots that were resolved, only 3 were significantly reduced in cells depleted of Alba proteins indicating that the effect on translation is highly specific. These spots were picked and their identities determined by mass spectrometry. Spot 3, which was present at very low levels, was identified as Alba2; this is consistent with our observations that it is also reduced upon knockdown of Alba3&4. Since the Alba2 polypeptide has a predicted pI of 9.3, the spot detected under the conditions used (first dimension pH 4–7) is likely to be a minor component that is modified post-translationally. Spots 1 and 2, which differed 1.82-fold and 1.62-fold between uninduced and induced cells, were both identified as GFP (with differing pI). In this cell line the GFP coding region has replaced one copy of the GPEET coding region and is flanked by the corresponding 5′ and 3′ UTRs, while EP3 has been replaced by the puromycin resistance gene. The reduction in GFP was confirmed by Western blot analysis (Figure S7). Once again, depletion of Alba proteins had little effect on mRNA levels (Figure 4E). Taken together, these results indicate that Alba proteins interact specifically with the GPEET 3′ UTR and stimulate translation, but this effect seems to be overridden by the open reading frame in the case of GPEET itself. A hierarchy of regulation, in which procyclin open reading frames are dominant and can prevent expression, has previously been observed in bloodstream and epimastigote (salivary gland) trypanosomes [21], [25].

### Functional diversification of eukaryotic Alba-domain proteins

Alba-domain proteins Rpp20/Pop7 and Rpp25/Pop6 are part of yeast and human RNase P/MRP complexes. RNase P and RNase MRP are related RNP complexes involved in a multitude of processes [46], [47], [48], [49], [50]. The main function of RNase P, however, is the removal of 5′ leader sequences from pre-tRNAs. Little is known about the processing of pre-tRNA 5′ ends in trypanosomatids. An RNase P/MRP RNA has remained elusive so far and, with the exception of Alba-domain proteins, no other proteins homologous to yeast and human subunits of the RNase P/MRP holoenzymes have been identified [51]. Ribonuclease P activity has been described in the mitochondrion of *T. brucei* but the proteins involved remain unknown [52]. A nuclear 300 kDa protein complex containing 2 putative methyltransferases and a La-like protein have been shown to be involved in the maturation of initiator methionyl-tRNA [53], [54]. To test the possibility that Alba proteins in *Trypanosoma brucei* are involved in pre-tRNA processing, we monitored Alba3&4 RNAi cells over a period of 12 days. As described above, in this cell line all four Alba proteins show reduced expression upon treatment with tetracycline. Total RNA was isolated at various time points, separated on polyacrylamide gels and used for Northern blot analysis. Two different probes were used for hybridisation, an oligonucleotide recognizing nucleotides 54–72 of isoleucine tRNA and an oligonucleotide complementary to nucleotides 47–64 of methionyl-initiator tRNA [55]. No differences could be seen between RNA samples from induced and uninduced RNAi cultures and a wild type control sample. In addition, no accumulation of precursor tRNAs was observed (Figure S9). Taken together these experiments indicate that Alba proteins in *T. brucei* do not participate in RNase P function despite their homology to known subunits of this RNA-protein complex from other eukaryotes.

### Alba proteins are localised in the cytoplasm and can be recruited to stress granules

**Figure 5:**
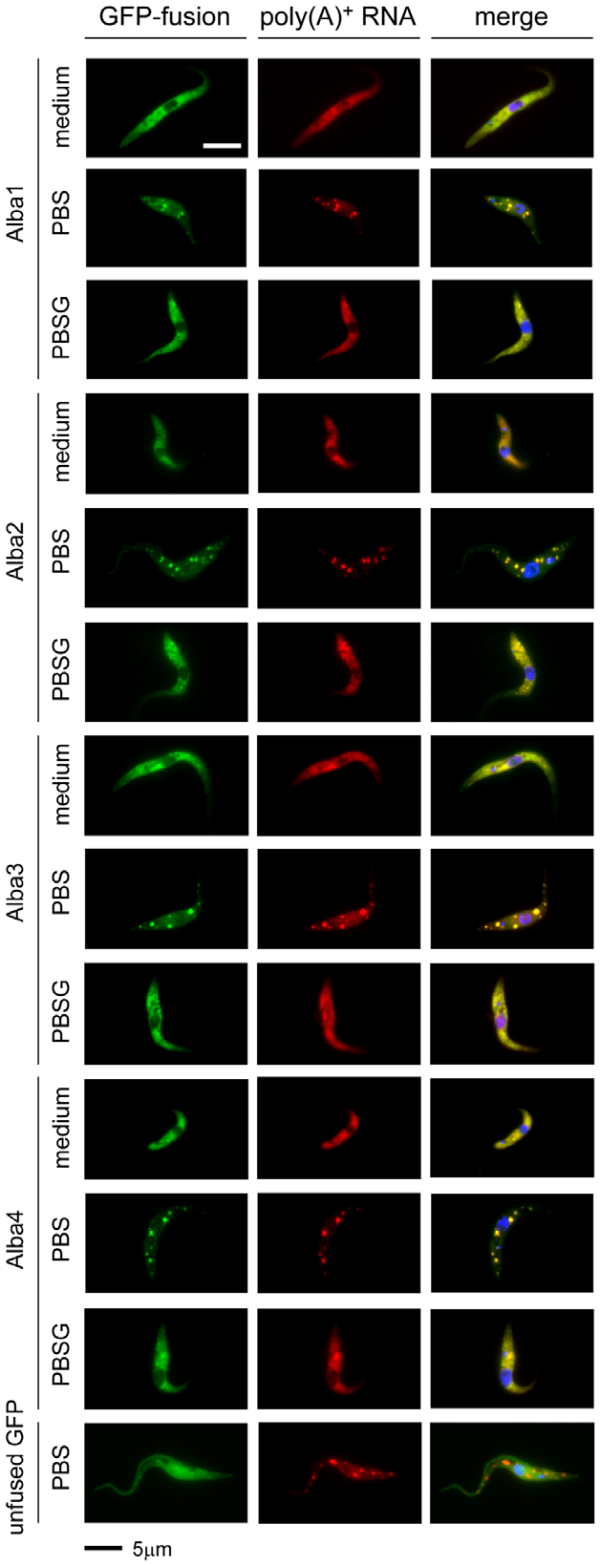
Alba proteins localise to the cell cytoplasm and become part of stress granules (SG) when cells are deprived of nutrients. Alba proteins were expressed as GFP fusion proteins. Cells expressing unfused GFP were used as a control. Poly(A) RNA was detected by fluorescence *in situ* hybridisation using a Cy3-labeled oligo(dT)_30_ probe. Cells were incubated for 2 h in PBS, PBS + glucose (PBSG) or in complete medium prior to fixation and analysis. Merge refers to the overlay of GFP and Cy3 signals in combination with signals from the DNA stain DAPI.

Human RNase P/MRP subunits Rpp20 and Rpp25 are mostly located in the cell nucleus. Proper localisation of Rpp20 was shown to be dependent on interaction with Rpp25 [44]. To investigate their localisation in *T. brucei,* we ectopically expressed GFP-tagged versions of all four Alba proteins. We observed a clear cytoplasmic localisation for the four proteins (Figure 5). Immunofluorescence experiments using Alba-specific antibodies showed the same localisation (Figure S10). Many proteins involved in RNA metabolism and translation have been described to be components of cytoplasmic ribonucleoprotein granules such as P bodies or stress granules (SG) [56], [57], [58], [59], [60]. Given the fact that Alba proteins bind RNA and, in the case of *Plasmodium berghei,* have been shown to copurify with protein complex(es) that have a granular distribution within the cell cytoplasm [61], we investigated if trypanosome Alba proteins were capable of translocating to SGs. Parasites expressing GFP-Alba fusion proteins were subjected to nutrient stress and poly(A) was detected by *in situ* fluorescence hybridisation (FISH) as described [62]. Upon stress, all Alba proteins concentrated in cytoplasmic SGs together with the poly(A) RNA (Figure 5). To check if SG formation was dependent on Alba proteins, we incubated Alba3&4 RNAi cells in the presence or absence of tetracycline. Cells were then subjected to nutrient stress and analysed by FISH as outlined above. In both cases SGs assembled normally, excluding a central function of Alba proteins in this process (Figure S11).

**Figure 6:**
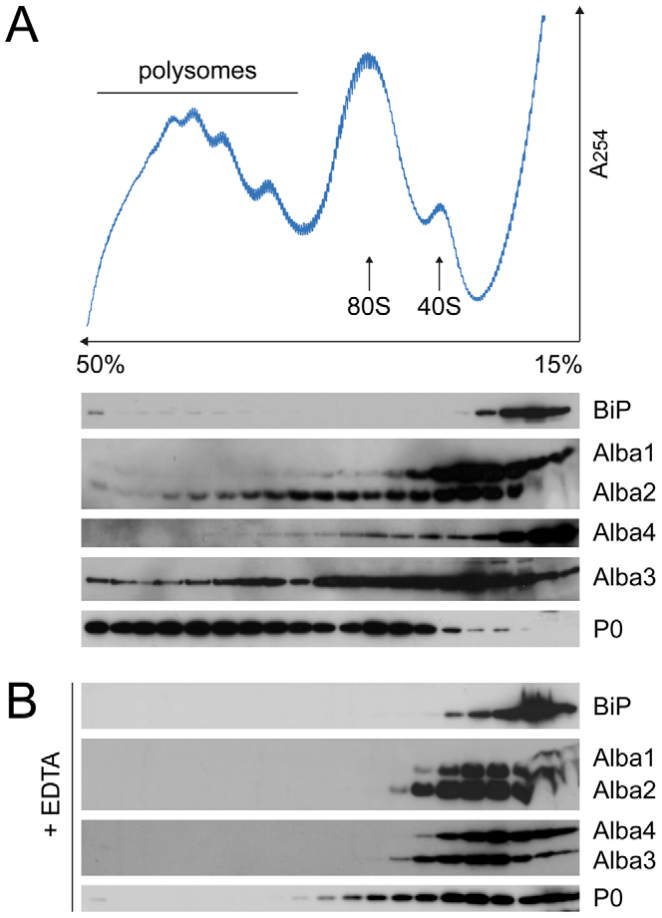
Alba2 and Alba3 partially associate with polysomes. (A) Protein extracts from wild type AnTat1.1 were separated on sucrose gradients. Absorption at 254 nm was recorded during fractionation of the gradient. Fractions were separated by SDS-PAGE and analyzed by immunoblotting. The ribosomal protein P0 served as a ribosome marker and the endoplasmic reticulum protein BiP as a marker for proteins not associated with ribosomes. (B) Fractionation of extracts after treatment with 50 mM EDTA, which disrupts polysomes.

Since Alba proteins specifically affected protein synthesis, we analysed their association with polysomes. An antibody recognizing the ribosomal stalk protein P0 served as a marker for ribosomes and an antibody directed against the ER chaperone protein BiP was used as a control for non-polysome-associated proteins. We found that Alba2 and Alba3 partially co-migrating with polysomes, and partially in the lighter fractions at the top of the gradient, while Alba1 and Alba4 were only detected in the lighter fractions (Figure 6A). When extracts were treated with 50 mM EDTA to disrupt ribosomes, P0 and Alba2 and Alba3 shifted towards the top of the gradient (Figure 6B).

### Tandem affinity purification and co-immunoprecipitation experiments provide insights into the Alba protein complexes

**Figure 7:**
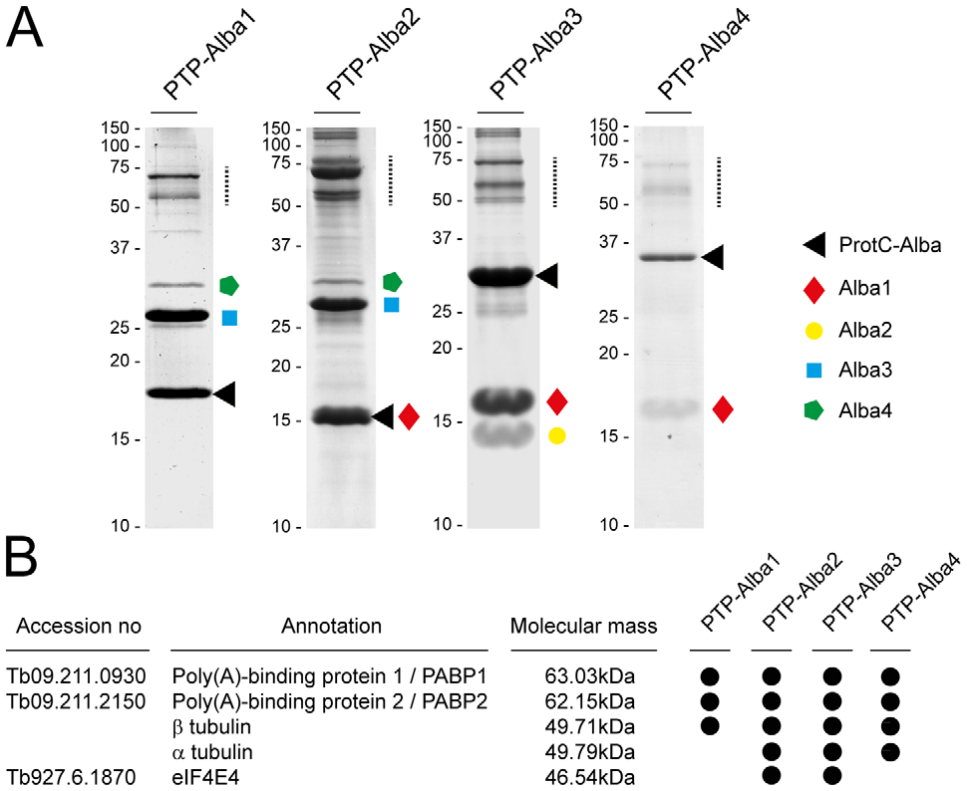
Tandem affinity purification (TAP) reveals interactions between Alba proteins. (A) Coomassie blue-stained polyacrylamide gels of purified complexes. Alba proteins were tagged *in situ* with a PTP tag and protein complexes isolated under native conditions. Proteins were treated with trypsin and the resulting peptides identified by LC-MS/MS. ProtC (black triangle) identifies the respective tagged Alba protein after removal of the protein A moiety with AcTev protease. Alba proteins co-purifying with the tagged proteins are highlighted as follows: Alba1 (red diamond), Alba2 (yellow circle), Alba3 (blue square) and Alba4 (green pentagon). (B) List of proteins that were identified independently in at least two TAP experiments in the size range indicated by the dashed lines in (A). α and β tubulin are likely to be contaminants as reported by others.

To identify components of the Alba complexes we established stable transformants in which one allele of the respective Alba gene was tagged *in situ* with an N-terminal PTP-tag for tandem affinity purification (TAP) [63]. Purified complexes were analysed by mass spectrometry. These TAP experiments revealed extensive interactions between the Alba proteins. The suspected associations between Alba3/Alba1 and Alba3/Alba2 could be confirmed. Although it was not detected as a component of the band shifts, Alba4 was found in sub-stochiometric amounts in complexes with Alba1 and Alba2. The poly(A)-binding proteins PABP1 and PABP2 were detected in all purifications. Eukaryotic translation initiation factor 4E4 (eIF4E4) was identified in complexes containing PTP-Alba2 and PTP-Alba3 (Figure 7A and B). α and β tubulin were also detected in three purifications. Their occurrence, however, most probably reflects contamination due to the high expression levels of these proteins. In TAP experiments with either PTP-Alba2 or PTP-Alba4 we were able to identify eIF4G3, eIF4A1 and eEF1A (data not shown).

**Figure 8:**
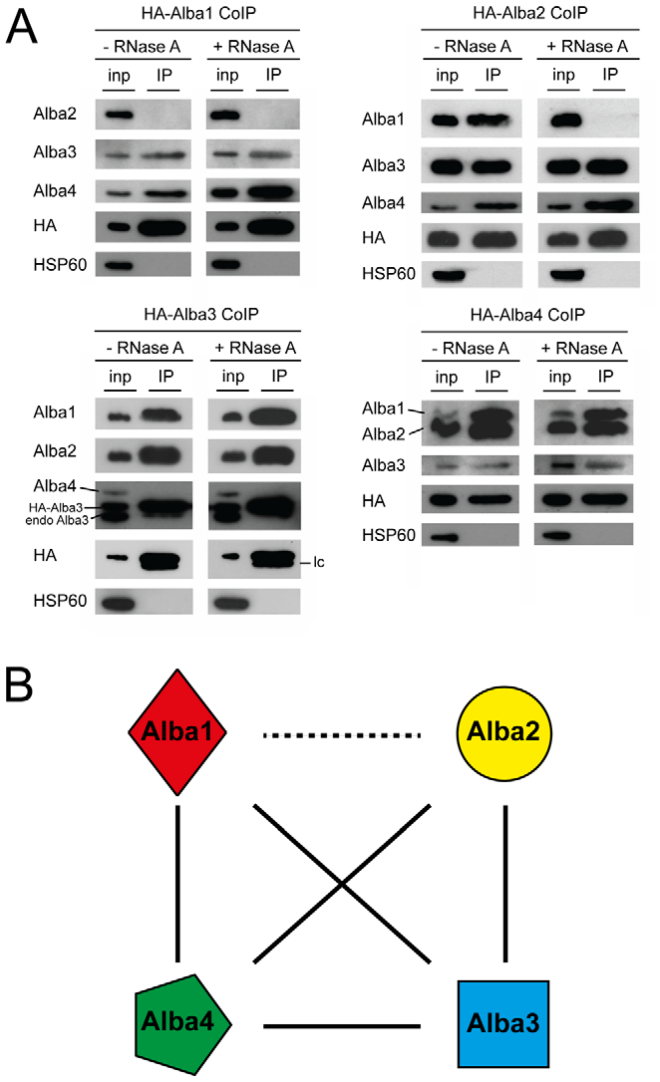
Interactions between Alba proteins are largely independent of bridging RNA molecules. (A) Ectopic expression of HA-tagged Alba proteins was induced by addition of tetracycline to cell cultures prior to extract preparation and CoIP. Input protein samples (inp) and precipitated proteins (IP) were analyzed by immunoblotting using Alba-specific antibodies. Detection of HA served as a positive control and HSP60 as a negative control for immunoprecipitation. Epitope-tagged Alba3 (HA-Alba3) and endogenous Alba3 (endo Alba3) cross-react with the bivalent anti-Alba4 antibody. lc: light chain of the anti-HA antibody used for the pulldown. (B) Summary of interactions between Alba proteins determined from TAP and CoIP experiments. Full black lines depict interactions that are resistant to RNase A. The dashed line indicates an RNase-sensitive interaction.

We further analysed the interactions between the *T. brucei* Alba proteins by performing co-immunoprecipitation (CoIP) experiments with ectopically expressed Alba proteins carrying N-terminal HA-tags. In this set of experiments we observed additional interactions of Alba4/Alba2 and Alba4/Alba3 with tagged Alba4 as bait (Figure 8A). This indicated that the PTP-tag, which has a size of ∼19 kDa [63] might have obstructed the interaction of Alba4 with some of its partners. CoIP of Alba1 and Alba2 was only detected when Alba2 was tagged, again suggesting interference by a tag on Alba1. Since Alba is a known nucleic acid-binding domain and, in addition, Alba3 and Alba4 contain RGG repeats known to occur in many RNA-binding proteins, we repeated the CoIP experiments with HA-tagged Alba proteins in the presence or absence of RNase A. However, with the exception of the Alba1/Alba2 interaction, no dependence on RNA was observed (Figure 8A). A summary of interactions between Alba proteins is presented in Figure 8B. In addition, we expressed truncated versions of Alba3 and Alba4 carrying N-terminal HA tags, but lacking the C-terminal RGG repeats. These experiments showed that association of Alba3 and Alba4 with other Alba proteins is independent of their C-terminal sequences (Figure S12). In summary, these experiments revealed that Alba proteins in *T. brucei* form at least one complex, or possibly several sub-complexes, with Alba3 likely to be a core component. Interactions between Alba proteins were shown to be largely RNA-independent and RGG repeats were dispensable for these interactions.

**Figure 9:**
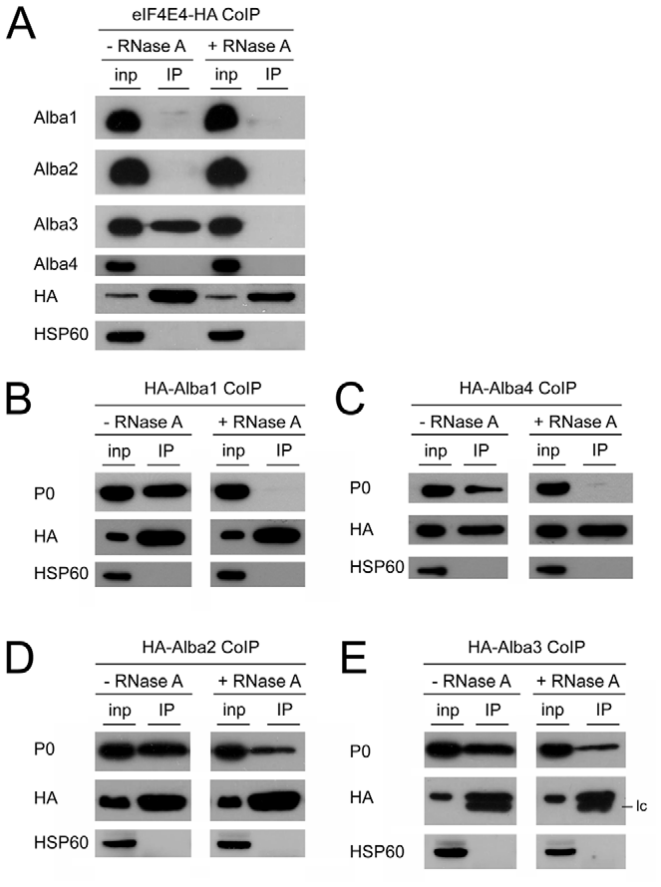
Alba proteins interact with the translation machinery. (A) Alba3 interacts with eIF4E4 via an RNA bridge. Expression of eIF4E4-HA was induced by addition of tetracycline. Extracts were either left untreated or treated with RNase A prior to performing CoIP. Protein input samples (inp) and precipitated proteins (IP) were analyzed by immunoblotting using Alba-specific antibodies. HA and HSP60 serve as controls for fusion protein expression and loading, respectively. (B–E). Ribosomal protein P0 co-precipitates with Alba proteins. CoIP experiments were performed using cells treated with tetracycline to induce expression of HA-tagged Alba proteins. Input protein samples (inp) and precipitated proteins (IP) were analyzed for the presence of the ribosomal protein P0. Detection of HA served as a positive control and HSP60 as a negative control for immunoprecipitation. HA-Alba1 (B) and HA-Alba4 (C) precipitate P0. Incubation with RNase A (10 µg/ml) abolishes these interactions. HA-Alba2 (D) and HA-Alba3 (E) also associate with P0 but treatment with 40 µg/ml RNase A does not completely abolish the interaction.

As mentioned above, the cap-binding protein eIF4E4 co-purified with Alba2 and Alba3 and eIF4G3 and eIF4A1 co-purified with Alba2 and Alba4, respectively. Together we identified all potential components of the trypanosome eIF4F complex - even though not in single purification experiments - along with PABP1/PABP2 which are known to be linked to eIF4F via eIF4G in other systems [64]. To consolidate these findings we expressed HA-tagged versions of eIF4E4 and eIF4G3 and used them in CoIP experiments. Using this system we could not detect interactions between eIF4G3 and Alba2 or any other Alba protein. PTP-tagged versions of eIF4G3 did also not co-precipitate any Alba proteins. It cannot be excluded, however, that the tags prevent the interaction. In contrast, the association between Alba3 and eIF4E4 could be confirmed. Furthermore, the Alba3/eIF4E4 co-precipitation could be blocked efficiently by treating the extract with RNase A, indicating the presence of RNA molecules that bridge the two proteins (Figure 9A). Treatment of trypanosomes with cycloheximide or puromycin prior to CoIP did not affect the interaction (data not shown). Finally, to test if Alba proteins interact with ribosomal proteins, we again performed CoIPs. The ribosomal stalk protein P0 was detected in precipitates of all four Alba proteins. Treatment of the extracts used for CoIP with 10 µg/ml RNase A disrupted the association of P0 with Alba1 and Alba4 (Figure 9B and C). In contrast, addition of up to 4-fold more RNase A was not sufficient to completely abolish the association of P0 with Alba2 and Alba3 (Figure 9D and E). These results confirm that the latter not only co-sediment with ribosomes, but are physically associated with them.

## Discussion

In this study we used the GPEETLII domain, which encompasses the GRE, to hunt for proteins regulating GPEET expression. Synthetic GPEETLII proved highly suitable for the detection of interacting proteins by EMSA; the binding was sequence-specific and depended on an intact GRE. Using this assay in combination with column chromatography led to the identification of 3 Alba domain proteins (Alba1, 2 and 3) that formed two complexes *in vitro*. Although highly similar to Alba3, the remaining member of the family, Alba 4, did not contribute detectably to the complexes seen by EMSA. Depletion of Alba proteins by RNAi had little effect on the steady state levels of GPEET mRNA, but decreased the translation of a GFP reporter transcript flanked by GPEET UTRs approximately 2-fold in procyclic forms cultured in the presence of glycerol. This is compatible with previous findings that the GRE is bifunctional and that, in addition to destabilising mRNA in late procyclic forms, it acts as a positive element that increases expression of a reporter gene approximately 2-fold in early procyclic forms. Taken together, this is consistent with Alba proteins acting via the GRE. Despite the presence of glycerol in the medium, we noted that GPEET expression was relatively low in the RNAi line and did not decrease further when RNAi was induced. Although glycerol retards shutdown of GPEET, it cannot prevent it indefinitely, and it can be overridden by other factors such as hypoxia [23]. Culture times of several weeks are unavoidable since it takes considerably longer to isolate clones of AnTat 90-13 than of more commonly used strains such as *T. brucei* 29-13. Another possible explanation for these differences is that elements in the procyclin coding regions add an additional layer of regulation. There is a precedent for this in the action of TbZFP3, a protein that binds both EP1 and GPEET 3′ UTRs, but only stimulates translation of EP1 [26].

Alba proteins are part of a superfamily that spans all three domains of life. For the most part, however, their functions remain obscure. Alba proteins were first identified as components of chromatin in *Sulfolobus,* but postulated to have additional functions on account of their structure and their ability to bind RNA [32], [65], [66], [67]. *Spo*VS, a bacterial protein with an Alba domain, is required for sporulation of *B. subtilis*, but its actual role has not been established [68]. The best-studied eukaryotic members of the family are the human and yeast proteins Rpp20/Pop7 and Rpp25/Pop6, which form heterodimers that bind the P3 stem of the RNA moiety in RNase P and RNase MRP [69], [70]. However, one study suggested that they are only transiently associated with RNase MRP *in vivo* and are not present in the catalytically active complex [71]. Phylogenetically, *T. brucei* Alba1 and Alba2 group with Rpp20/Pop7 and Alba3 and Alba 4 with Rpp25/Pop6, and in common with many other members of the latter family, Alba3 and Alba4 also contain RGG repeats at their C-termini.

Knockdown of Alba3&4 by RNAi, (which also results in depletion of Alba1 and Alba2) had no effect on tRNA processing. Moreover, in contrast to the human and yeast proteins, which are nuclear, trypanosome Alba proteins are cytoplasmic. Alba3 appears to occupy a pivotal position in procyclic forms – it is present in both complexes formed with GPEETLII *in vitro*, it co-purifies in stoichiometric amounts with Alba1 or Alba2, and it is either required for their translation or their stability *in vivo*. In addition, it is the only Alba protein whose depletion causes a growth phenotype. Based on tandem affinity purification and CoIP all four Alba proteins are present in mRNPs that contain the poly(A) binding proteins PABP1 and PABP2. The fact that the interaction between Alba 1 and Alba2 is destroyed by RNase suggests that Alba1/Alba3 and Alba2/Alba3 might exist as subcomplexes that are bridged by RNA. Alba2 and Alba3 are also more closely linked to the translation machinery than the other two proteins. All four Alba proteins co-precipitate with the ribosomal protein P0, but the interaction with Alba1 and Alba4 is RNase-sensitive. In contrast, the interaction with Alba2 and Alba3 is partially resistant and these two proteins co-migrate with polysomes in sucrose gradients. In addition, Alba3 co-precipitates with the cap-binding protein eIF4E4, although this interaction may be indirect as it is also susceptible to RNase treatment.

Since Alba proteins and eIF4E4 are moderately abundant (∼10^4^ copies per cell; our unpublished data and [72]) and exceed the number of procyclin transcripts by an order of magnitude, we considered that they might be more general regulators of translation. As judged by 2D-DIGE, however, their effect on translation is highly specific. Of 2300 spots resolved after knockdown of Alba3&4, one spot identified as Alba 2 and 2 spots identified as the GFP reporter from the GPEET locus were the only proteins to be significantly affected. It cannot be excluded that some proteins with isoelectric points outside the range of this analysis (including the highly basic Alba proteins), or proteins that are expressed at very low levels, or even alterations in post-translational modifications might have escaped detection. Indeed, this seems quite likely to be the case, given that the cells grew more slowly when they were depleted of Alba proteins. Alba proteins are not the only example of RNA-binding proteins that outnumber their target RNAs in trypanosomes. A striking example is DHH1, which exceeds the total number of mRNA molecules per cell by a factor of ten, but is nevertheless specific for developmentally regulated transcripts [73].

At present we do not know if Alba proteins have additional functions in other stages of the trypanosome life cycle. They appear to be constitutively expressed in bloodstream forms and early and late procyclic forms, but it is possible that stage-specific post-translational modifications modulate their activity. Phosphorylated forms of Alba2 and Alba3 have been detected in phosphoproteome analyses [74], [75]. The acronym Alba (acetylation lowers binding affinity) for the archaeal proteins refers to acetylation of lys16, and its deacetylation by SIR2 [76]. Although this residue is not conserved in the trypanosome Alba proteins, and the SIR2-like proteins are not cytoplasmic [77], we cannot rule out modifications of other residues by other enzymes.

Recently, Alba proteins, PABP and eIF4E were found to co-purify with two proteins from gametocytes of *Plasmodium berghei*, DOZI (the orthologue of *T. brucei* DHH1), and CITH (the orthologue of *T. brucei* Scd6). Since these proteins localise to P granules in the cytoplasm of female gametocytes and have been implicated in stabilising and translationally silencing maternal mRNAs, the authors suggested that Alba proteins might also contribute to translational repression [61]. In trypanosomes, all four Alba proteins are recruited to starvation granules, as are DHH1 and PABP [62]. However, Alba complexes purified from unstressed cells contain neither Scd6 nor DHH1, nor do Alba proteins co-purify with Scd6 (BS, MH and IR, manuscript in preparation). While Alba proteins in *Plasmodium* and trypanosomes may have divergent functions, we nevertheless consider it possible that that the Alba proteins in *Plasmodium* are not acting as translational repressors. Instead, they might be sequestered in the P granules together with their target mRNAs, and be released together for translation.

## Materials and Methods

### Ethics statement

Immunisation of animals was performed at the Central Animal Facility of the Institute of Pathology, Inselspital, Bern. The procedure was approved by the local veterinary authorities (Veterinäramt, Kanton Bern) in compliance with Swiss federal law (TSchG) and cantonal by-laws (TSchV Bern).

### Cell culture

Procyclic forms of the following strains of *T. brucei* were used in this study: AnTat 1.1 [78] and the derivatives AnTat 90-13 [36] and AnTat 90-13/GFP-GPEET (G. Schumann Burkard, manuscript in preparation) for RNAi experiments and inducible expression of HA-tagged proteins. Cells were cultured at 27°C in DTM supplemented with 15% heat-inactivated fetal bovine serum (FBS) [79] or SDM-79 [80] supplemented with 10% FBS and 20 mM glycerol. Stable transformations of parasites were performed as described previously [81].

### Constructs and primers

A list of DNA constructs, primers and restriction sites used for cloning can be found in the supplementary information (Table S1). Genomic DNA from *T. brucei* AnTat 1.1 or DNA constructs derived from it was used as a template for PCR. All constructs were verified by sequencing.

#### Bacterial expression constructs

Open reading frames (ORF) of Alba1-4 were cloned into bacterial expression vectors pMBP-Parallel2 or pMBP-Parallel3 [82] to allow expression of fusion proteins in *E. coli*.

#### RNAi constructs

For RNAi constructs the stem-loop vector pSLComp1 was used. This vector, which is based on pNA8 [83], was adapted to contain two pairs of compatible restriction sites (BclI/BamHI and SalI/XhoI) flanking a stuffer region to allow sequential ligation of RNAi fragments in opposite orientations. For Alba1 and Alba2 RNAi, full-length ORFs were used for vector construction. For Alba3- and Alba4-specific constructs, unique sequences from their respective 3′ UTRs were chosen. The Alba1&2 double RNAi vector was constructed by amplifying Alba1 and Alba2 ORFs with the same primer pairs used for the single RNAi constructs. The Alba1 fragment was digested with BamHI and the Alba2 fragment with BglII. The fragments were ligated, leading to an Alba2-Alba1 fusion fragment which was then amplified with flanking primers and cloned into pSLComp1. For Alba3&4 double RNAi, the full length Alba3 ORF was used. Due to the high sequence identity of the Alba3 and Alba4 genes this construct efficiently targets both mRNAs.

#### GFP-tagging constructs

ORFs of Alba1-4 were cloned into the GFP expression vector pG-EGFP-ΔLII [84] to express the Alba proteins as N-terminal GFP fusion proteins.

#### HA-tagging constructs

ORFs were amplified by PCR with forward primers including sequences encoding an HA tag. DNA fragments were cloned into the inducible expression vector pLEW100 [85]. For HA-Alba3-RGG and HA-Alba4-RGG constructs, reverse primers were designed to omit the C-terminal regions containing the RGG repeats of the respective proteins. For the eIF4E4-HA construct the pLEW100 expression vector was digested with HindIII and BamHI. Oligonucleotides P22 and P23 encoding an HA tag and containing flanking BamHI and AgeI sites were annealed and ligated into the vector keeping the HindIII site intact and destroying the original BamHI site. Using the HindIII and BamHI sites, an eIF4E4 PCR product was ligated in front of the sequence encoding the HA tag.

#### PTP constructs

*In situ* tagging of Alba proteins was performed using the vector pN-PURO-PTP [63]. Alba1 and Alba2 each contain a unique restriction site (NruI and ClaI, respectively) that enables targeting of endogenous alleles of the respective genes. For Alba3 and Alba4 a silent mutation generating a unique NcoI restriction site was introduced by overlap extension PCR. In the case of Alba3, primer pairs P30/P31 and P32/P33 and in the case of Alba4, primer pairs P34/P31 and P32/P33 were used to amplify respective DNA fragments. The two amplicons were then used for overlap extension PCR with the respective flanking primers.

### *In vitro* transcription and electromobility shift assay (EMSA)

Template DNA for *in vitro* transcription of RNA was amplified from plasmids pCAT-GPEET, pCAT-EPLII and pCAT-GPEETM234 [23], [24] using primer pairs T1/T2, T3/T4 and T5/T6 (Table S2). Primers were designed to include nucleotides 121–185 of the GPEET 3′ UTR and nucleotides 100-183 of the EP1 3′ UTR. Sense primers T1, T3 and T5 included the T7 RNA polymerase promoter sequence. For *in vitro* transcription of radioactively labeled GPEETLII RNA, 100 ng of purified template DNA was supplemented with 0.5 mM rATP, rCTP, rGTP, 50 nM rUTP, 1 MBq of [α-^32^P]-UTP (3000 Ci/mmol; Hartmann). T7 RNA polymerase (Roche) was used for transcription (2 h, 37°C). The reaction was treated with DNase I and purified over a Sephadex G50 column. Transcription of unlabeled competitor RNAs used 5 times more template DNA in a correspondingly bigger reaction volume. rATP, rCTP, rGTP and rUTP concentrations were adjusted to 1 mM each. Incorporation of radiolabel into GPEETLII was monitored by liquid scintillation counting and the concentration of transcripts measured by spectrophotometry. To generate protein extracts for band shift experiments, 5×10^7^ trypanosomes were harvested by centrifugation and washed in PBS. The cell pellet was dissolved in 100 µl CE lysis buffer (20 mM Tris-HCl pH7.6, 60 mM KCl, 1 mM EDTA, 1 mM DTT, 0.1% NP-40, Complete protease inhibitor EDTA-free (Roche)) and incubated on ice (5–10 min). The lysate was centrifuged at 16,000 g (15 min, 4°C). The resulting supernatant was supplemented with 12.5 µl 87% glycerol. For each binding reaction a reaction mix consisting of 1 µl (∼2 ng RNA, 30′000 cpm) labeled GPEETLII RNA, 2 µl binding buffer (100 mM Tris-HCl pH 7.6, 25 mM MgCl_2_, 7.5 mM KCl, 37.5 mM NaCl, 10 mM DTT, Complete protease inhibitor EDTA-free), 2 µl sucrose loading buffer (40% sucrose, 0.25% bromphenol blue, 0.25% xylene cyanol) and 2 µl H_2_O or competitor RNA was prepared. Unspecific binding was competed by addition of 1 µg yeast tRNA (Sigma) per reaction. 40 U RNase inhibitor (Roche) was added to an equivalent of 70 µl of reaction mix. In competition experiments, 200 ng of unlabeled GPEETLII, EPLII or GPEETM234 RNA were added to the reaction. Seven µl of the reaction mix was supplemented with 3 µl of protein extract and incubated for 30 min on ice. Samples were separated on 10% native polyacrylamide gels. Labeled RNA was detected by autoradiography.

### Protein purification

10^10^ trypanosomes were harvested by centrifugation and washed twice in PBS. The cell pellet was resuspended in 10 ml CE lysis buffer and incubated 10 min on ice. The extract was cleared by centrifugation at 8,000 g (15 min, 4°C). From this, a S100 was prepared by ultracentrifugation at 100,000 g (1 h, 4°C). The S100 was passed sequentially over two 1 ml HiTrap Heparin HP columns (GE Healthcare) equilibrated with chromatography buffer (20 mM Tris-HCl pH 7.6, 60 mM KCl, 1 mM EDTA, 1 mM DTT) connected to an FPLC system (Pharmacia). Proteins bound to the columns were eluted by a step gradient of 0.2 M, 0.5 M and 1 M KCl. Protein concentration was assessed for each fraction using the Coomassie Plus protein assay kit (Pierce) and RNA binding activity was tested by EMSA. Protein fractions active in band shift assays eluted at 350 mM KCl. Active fractions were pooled and subjected either to ion-exchange or size exclusion chromatography (SEC). For ion-exchange chromatography pooled fractions were diluted in salt-free buffer (20 mM Tris-HCl pH 7.6, 1 mM DTT, 1 mM EDTA) and loaded onto a MonoQ HR5/5 column (Pharmacia) equilibrated with chromatography buffer. Proteins bound to the matrix were eluted with a linear gradient from 60 mM to 1 M KCl. Active fractions eluted in the range of 250–350 mM KCl. SEC was performed using a Superdex 200 HR 10/30 column (Pharmacia). The column was calibrated with a protein size standard composed of ferritin (440 kDa), aldolase (158 kDa), BSA (67 kDa), ovalbumin (43 kDa), chymotrypsin (25 kDa) and lactalbumin (14.2 kDa). Pooled fractions from the HiTrap column were concentrated and washed with salt-free buffer prior to SEC. Separation of proteins was carried out in chromatography buffer. Active fractions eluted in a mass range from 115–33 kDa. Active fractions from both procedures were separated on SDS-PAGE gels. Protein bands that correlated with the band shift pattern were either subjected to Edman sequencing or digested with trypsin and analyzed by LC-MS/MS.

### Antibody production

Alba proteins were expressed as N-terminal MBP fusion proteins in *E. coli* BL21 (Stratagene). Bacteria were grown in LB medium supplemented with 0.2% glucose at 37°C. Expression was induced for 3 h with 1 mM IPTG. Cells were pelleted by centrifugation and resuspended in MBP buffer (20 mM Tris-HCl pH 7.6, 200 mM NaCl, 1 mM EDTA, 1 mM DTT and protease inhibitor EDTA-free). Cells were lysed by sonication and extracts cleared by centrifugation at 12,000 g (15 min, 4°C). Fusion proteins were bound to amylose affinity resin (New England Biolabs) and either eluted by cleavage with AcTev (Invitrogen) or by addition of 10 mM maltose. For immunisation of rabbits, 100 µg of the respective recombinant protein was mixed with GERBU Adjuvant 100 (Gerbu). Immunisation was repeated after two, four and six weeks. Rabbits were bled and the sera prepared according to standard procedures. In the case of immunisation with recombinant Alba4 a bispecific serum recognizing Alba4 and Alba3 resulted due to the high sequence identity of the two proteins. Specific antibodies were enriched by affinity purification using the recombinant proteins.

### Immunoblotting

The following primary antibodies were used for immunoblotting: polyclonal rabbit anti-Alba1, -Alba2, -Alba3 and -Alba4 (1∶500) antisera; rabbit anti-GPEET (1∶1,000)[86], mouse anti-GFP (1∶5,000; Roche), rat anti-HA (1∶1,000; Roche), mouse anti-Protein C coupled to HRP (Roche), mouse anti-HSP60 (>1∶10,000; [87] kindly provided by André Schneider, University of Bern), anti-BiP (1∶50,000; kindly provided by Jay Bangs, University of Wisconsin) and rabbit anti-TcP0 (1∶5,000; kindly provided by Keith Matthews, University of Edinburgh) [88]. HRP-coupled secondary antibodies (Dako) were used at a dilution of 1∶3,000.

### Tandem affinity purification (TAP)

For purification of PTP-tagged Alba proteins 2–3×10^10^ trypanosomes were harvested by centrifugation and washed twice with 100 mM NaCl, 3 mM MgCl_2_, 20 mM Tris-HCl pH 7.7 followed by a wash in buffer E (150 mM sucrose, 20 mM potassium glutamate, 20 mM Tris-HCl pH 7.7, 3 mM MgCl_2_, 0.5 mM DTT, Protease inhibitor EDTA-free). The cell pellet was then resuspended in 10 ml buffer E and cells were broken by douncing and freezing in liquid N_2_. One-tenth volume of 10x extraction buffer (1.5 M KCl, 20 mM Tris-HCl pH 7.7, 3 mM MgCl_2_, 0.5 mM DTT, 1% Tween-20) was added to the suspension and incubated on ice for 20 min. The extract was cleared twice by centrifugation at 16,000 g (15 min, 4°C) The supernatant was supplemented with Complete protease inhibitor EDTA-free (Roche) and subjected to tandem affinity purification according to Schimanski et al. [63]. Input, TEV eluate and EGTA eluate samples were separated on 14–16% SDS-PAGE gels and stained with blue silver colloidal Coomassie [89]. Protein bands were cut from gels, digested with trypsin and analysed by LC-MS/MS.

### Co-immunoprecipitations (CoIP)

Expression of HA-tagged proteins was induced for 1 to 2 days by addition of tetracycline (1 µg/ml) to the medium. For each experiment 5×10^8^ cells were harvested by centrifugation and washed twice in PBS. Cell pellets were resuspended in 500 µl IP buffer (20 mM Tris-HCl pH 7.6, 150 mM NaCl, 1 mM EDTA, 1 mM DTT, 0.5% NP-40, Complete protease inhibitor EDTA-free, RNase inhibitor (40 U/ml, Roche)). In case of RNase A treatment, the RNase inhibitor was replaced with 5–20 µg of RNase A. The extract was passed 5 times through a 27 gauge needle, incubated on ice for 10 min and centrifuged at 16,000 g (5 min, 4°C). From the resulting supernatant, 50 µl were withdrawn and mixed with 12.5 µl of 5X SDS loading buffer to serve as input sample. The remaining supernatant was incubated with 50 µl of anti-HA affinity matrix (Roche) for 2 h on a rotator at 4°C. Subsequently the HA beads were washed 5X in IP buffer. 95 µl NTE buffer (10 mM Tris-HCl pH 7.6, 100 mM NaCl, 5 mM EDTA) and 5 µl 10% SDS were added and HA beads incubated at 65°C (10 min). Beads were pelleted and the resulting eluate mixed with 25 µl of 5x SDS loading buffer, giving the IP sample. For immunoblotting 4 µl of the input sample and 16 µl of the IP sample were analysed.

### Polysome profiling

10^9^ log phase cells were harvested, washed twice in PBS and resuspended in 750 µl polysome lysis buffer (20 mM Tris-HCl pH 7.6, 120 mM KCl, 2 mM MgCl2, 1.2% NP-40, 1 mM DTT, Complete protease inhibitor EDTA-free, RNase inhibitor (40 U/ml)) and passed 5 times through a 27 gauge needle. The extract was cleared by centrifugation at 16,000 g (5 min, 4°C). 650 µl of the lysate was layered onto a linear 15–50% sucrose gradient (12 ml) prepared in polysome buffer (20 mM Tris-HCl pH 7.6, 120 mM KCl, 2 mM MgCl2, 1 mM DTT, Complete protease inhibitor EDTA-free, RNase inhibitor (40 U/ml)) and centrifuged at 40,000 rpm in a Beckman SW40 rotor (2.5 h, 4°C). Absorption was monitored at 254 nm using a UV-1 optical unit (Pharmacia) connected to a UV-1 control unit (Pharmacia) and a REC 102 recorder (Pharmacia). A P1 pump (Pharmacia) combined with a GradiFrac collector (Pharmacia) was used for fractionation. 50 mM EDTA was used to dissociate polysomes. Proteins were precipitated with StrataClean resin (Stratagene) for analysis by immunoblotting.

### Isolation of RNA and Northern blotting

Total RNA was extracted by the hot phenol [90] or guanidine thiocyanate method [91]. Northern blotting of agarose and urea gels was done as described [55], [90]. GPEET mRNA was detected using a DNA probe recognizing the internal repeat region [24]. Radioactive labeling was performed using the Megaprime DNA labeling system (Amersham Biosciences). 18S rRNA, isoleucine and methionyl-initiator tRNAs were detected with 5′ labeled oligonucleotides as described previously [55], [92], [93].

### Fluorescence *in situ* hybridisation (FISH) and fluorescence microscopy

FISH of poly(A) RNA was carried out as described [62]. Slides were mounted with VectaShield containing 4,6-diamidino-2-phenylindole (DAPI) (Vector Laboratories Inc.). Cells subjected to nutrient deprivation were harvested by centrifugation, washed in PBS or PBSG (PBS, D-glucose (2.3 g/l)), resuspended in a volume of PBS or PBSG corresponding to the original culture and incubated at 27°C for the periods indicated. Images were acquired with a DFC350 FX monochrome CCD camera (Leica) mounted on a DM6000B microscope (Leica). Images were superimposed and analysed using LAS AF software (Leica).

### Phylogenetic tree

Amino acid sequences corresponding to Alba domains were extracted from Alba proteins of various species using Pfam [94]. Sequences were aligned with the ClustalW2 algorithm using Seaview 4.2.4 software [95]. A parsimony-based phylogenetic tree was built ignoring all gap sites and performing 100 bootstrapping replicates using Seaview.

### Mass spectrometry of procyclins

Mass spectrometry was carried out according to Walrad et al. [26]. Briefly, freeze-dried parasites were extracted with chloroform/methanol/water, 10∶10∶3 (v/v/v) and centrifuged to remove lipids. Pellets were then extracted with 9% butanol. The butanol fractions containing the procyclins were freeze-dried and dephosphorylated by treatment with 48% aqueous hydrofluoric acid. Dephosphorylated samples were freeze-dried and washed with water, followed by incubation in 40 mM TFA at 100°C. Equal amounts of each sample were mixed with a-cyano and negative-ion MALDI-TOF spectra were acquired.

### 2D-DIGE analysis

The analysis was performed following the manufacturer's recommendations (GE Healthcare) and as previously described by Morales et al. [96]. Briefly, total protein extracts were obtained from four independent cultures of *T. brucei* Alba 3&4 RNAi, grown for four days in the presence (+Tet) or absence (-Tet) of 1 µg/ml tetracycline. Following precipitation and resolubilisation, equal amounts of protein were differentially labelled with the spectrally resolvable Cy3 and Cy5, mixed with a Cy2 labeled internal control, and samples were separated in the first dimension using the IPGphor isoelectric focusing system (GE Healthcare) and 18 cm DryStrip pH 4-7 Immobiline strips. After equilibration, strips were transferred to SDS polyacrylamide gels and separated in the second dimension using 12.5% SDS-PAGE gels and an Ettan DALT six electrophoresis system (GE Healthcare). Gels were scanned on a Typhoon Variable Mode Imager 9400 (GE Healthcare) and quantitative analysis of the images was performed using the DeCyder 2D Differential Analysis Software (GE Healthcare). Spots showing a minimum of 1.5-fold change in fluorescence intensity with a p-value <0.01 were considered significantly modulated and subjected to MS determination on the 4800 Proteomics Analyzer (Applied Biosystems, USA). Protein hits with MASCOT Protein score ≥ 50 and a GPS Explorer Protein confidence index ≥ 95% were used for further manual validation.

## Supporting Information

Figure S1 **Sequence alignment of** ***in vitro*** **transcribed GPEET and EP LII RNAs used for band shift assays.** The region corresponding to the glycerol responsive element (GRE) of GPEET is indicated. In GPEETM234 nucleotides in the GRE are mutated to the corresponding sequences in EP.
(TIF)

Figure S2 **ClustalW multiple sequence alignment of** ***Trypanosoma brucei*** **Alba proteins.** The consensus sequence and sequence logo are indicated above the alignment. The blue bar indicates the approximate position of the Alba-domains. The magenta bar indicates the carboxy-termini of Alba3 and Alba4 containing RGG repeats. The FDXh and GYQXP motifs characteristic for the Rpp20/Pop7 and Rpp25/Pop6 Alba subfamilies are highlighted in green and red, respectively. X is any amino acid and h corresponds to hydrophobic amino acids. The red triangle indicates the highly conserved glycine residue.
(TIF)

Figure S3 **Knockdown of Alba3 or Alba3&4 slows growth.** Growth of (A) Alba3 and (B) Alba3&4 RNAi cells in DTM + 15% FBS was monitored for 10 days without induction (- Tet, black circles) or with induction by tetracycline (+ Tet, black squares). Cells were counted and diluted to 3×10^6^ ml^−1^ daily. Graphs show the cumulative cell number per ml medium.
(TIF)

Figure S4 **MRP1 and MRP2 interact with GPEETLII RNA** ***in vitro*** **.** Protein extracts from uninduced (- Tet) and induced (+ Tet) cultures of MRP1&2 RNAi were incubated in the presence of ^32^P-GPEETLII RNA followed by separation on 10% native polyacrylamide gels. Ablation of MRP1 and MRP2 led to marked reduction of the S1 band shift compared to the control cells after 3 days of induction.
(TIF)

Figure S5 **Knockdown of Alba1 and Alba2 does not affect Alba3 protein levels.** Western blots using antibodies specific for Alba1, Alba2 and Alba3 were performed with protein samples from uninduced (- Tet) and induced (+ Tet) cells. (A) Alba1, (B) Alba2, (C) Alba1&2 RNAi. HSP60 served as loading control.
(TIF)

Figure S6 **Northern blot analysis of GPEET mRNA during differentiation of early procyclic forms to late procyclic forms.** (A) Total RNA was isolated at the times indicated after removal of glycerol from the medium. Hybridisation conditions and probes for GPEET and 18S were as for Figure 8. (B) Quantification of signals after normalisation to 18S rRNA.
(TIF)

Figure S7 **Comparison and quantification of GPEET and GFP protein levels in uninduced and induced cultures of Alba3&4 RNAi cells.** (A) Western blots were performed with the appropriate antibodies (see Materials & Methods). (B) GPEET and GFP levels were normalised against HSP60.
(TIF)

Figure S8 **Analysis of procyclin isoforms after knockdown of Alba3.** Procyclins were analysed in uninduced (- Tet) and tetracycline-induced (+ Tet) Alba3 RNAi cells over a period of 8 days. Negative ion MALDI-TOF spectra show the major procyclin isoforms. GPEET expression varies in culture, but is not affected by knockdown of Alba3. C1 -C4 denote independent cultures.
(TIF)

Figure S9 **Downregulation of Alba proteins does not lead to accumulation of tRNA precursors.** (A) RNA from wild type AnTat1.1 (wt) and Alba3&4 RNAi cells cultured in the presence (+Tet) or absence (-Tet) of tetracycline was separated on polyacrylamide gels and stained with ethidium bromide. Representative RNA species are indicated on the left. Northern blots were performed with probes recognizing methionyl initiator (Met_i_) tRNA (B) and isoleucine (Ile) tRNA (C).
(TIF)

Figure S10 **Immunofluorescence analysis of Alba1, Alba2 and Alba3.** Alba1, Alba2 and Alba3 were detected with specific antibodies as indicated. Cells were either incubated for 3 h in PBS or in PBS + glucose (PBSG). The scale bar represents 10 mm. DIC: Differential interference contrast. DAPI: 4,6-diamidino-2-phenylindole. Samples were analysed with a Leica DM IRE2 inverted microscope connected to a Leica True Confocal Scanner.
(TIF)

Figure S11 **Alba proteins are dispensable for stress granule (SG) formation.** Alba3&4 RNAi cells were cultured for 3 days in the presence (+ Tet) or absence (- Tet) of tetracycline. Knockdown of Alba 3&4 also causes depletion of Alba 1 and Alba2. Parasites were stressed by incubation in PBS for 3 h or kept in normal medium. Poly(A) RNA was detected by fluorescence *in situ* hybridisation using a Cy3-labeled oligod(T)_30_ probe. Nuclei and kinetoplasts were visualized by DAPI staining.
(TIF)

Figure S12 **RGG repeats of Alba3 and Alb4 are dispensable for interactions with Alba proteins and P0.** (A) HA-tagged Alba3 and Alba4 lacking the carboxy-terminal RGG repeats (shown in red) were ectopically expressed. (B) Protein samples corresponding to the input (inp) and precipitated (IP) material were analyzed by immunoblotting with the antibodies indicated. Anti-HA served as a positive control for the pulldown and HSP60 as a negative control. lc indicates signals from the light chain of the anti-HA antibody used for the pulldown.
(TIF)

Table S1 **List of primers and constructs used for cloning.**
(XLS)

Table S2 **List of primers used for** ***in vitro*** **transcription of RNA.**
(XLS)
