# Supplementary figures and images for: Alba-Domain Proteins of Trypanosoma brucei Are Cytoplasmic RNA-Binding Proteins That Interact with the Translation Machinery
Source: PLoS One. 2011 Jul 21;6(7):e22463. doi: 10.1371/journal.pone.0022463 (PMC3141063; doi:10.1371/journal.pone.0022463)

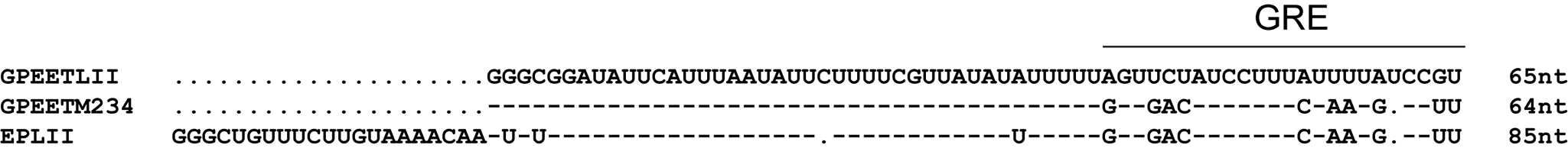

Supplement: Figure S1 — Sequence alignment of in vitro transcribed GPEET and EP LII RNAs used for band shift assays. The region corresponding to the glycerol responsive element (GRE) of GPEET is indicated. In GPEETM234 nucleotides in the GRE are mutated to the corresponding sequences in EP. (TIF) [file pone.0022463.s001.tif]

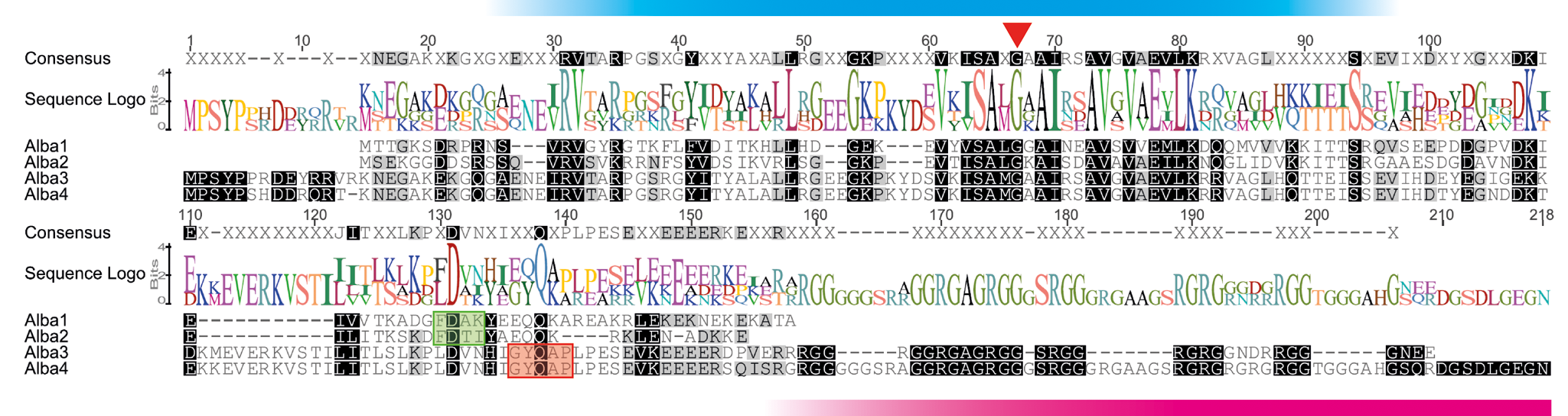

Supplement: Figure S2 — ClustalW multiple sequence alignment of Trypanosoma brucei Alba proteins. The consensus sequence and sequence logo are indicated above the alignment. The blue bar indicates the approximate position of the Alba-domains. The magenta bar indicates the carboxy-termini of Alba3 and Alba4 containing RGG repeats. The FDXh and GYQXP motifs characteristic for the Rpp20/Pop7 and Rpp25/Pop6 Alba subfamilies are highlighted in green and red, respectively. X is any amino acid and h corresponds to hydrophobic amino acids. The red triangle indicates the highly conserved glycine residue. (TIF) [file pone.0022463.s002.tif]

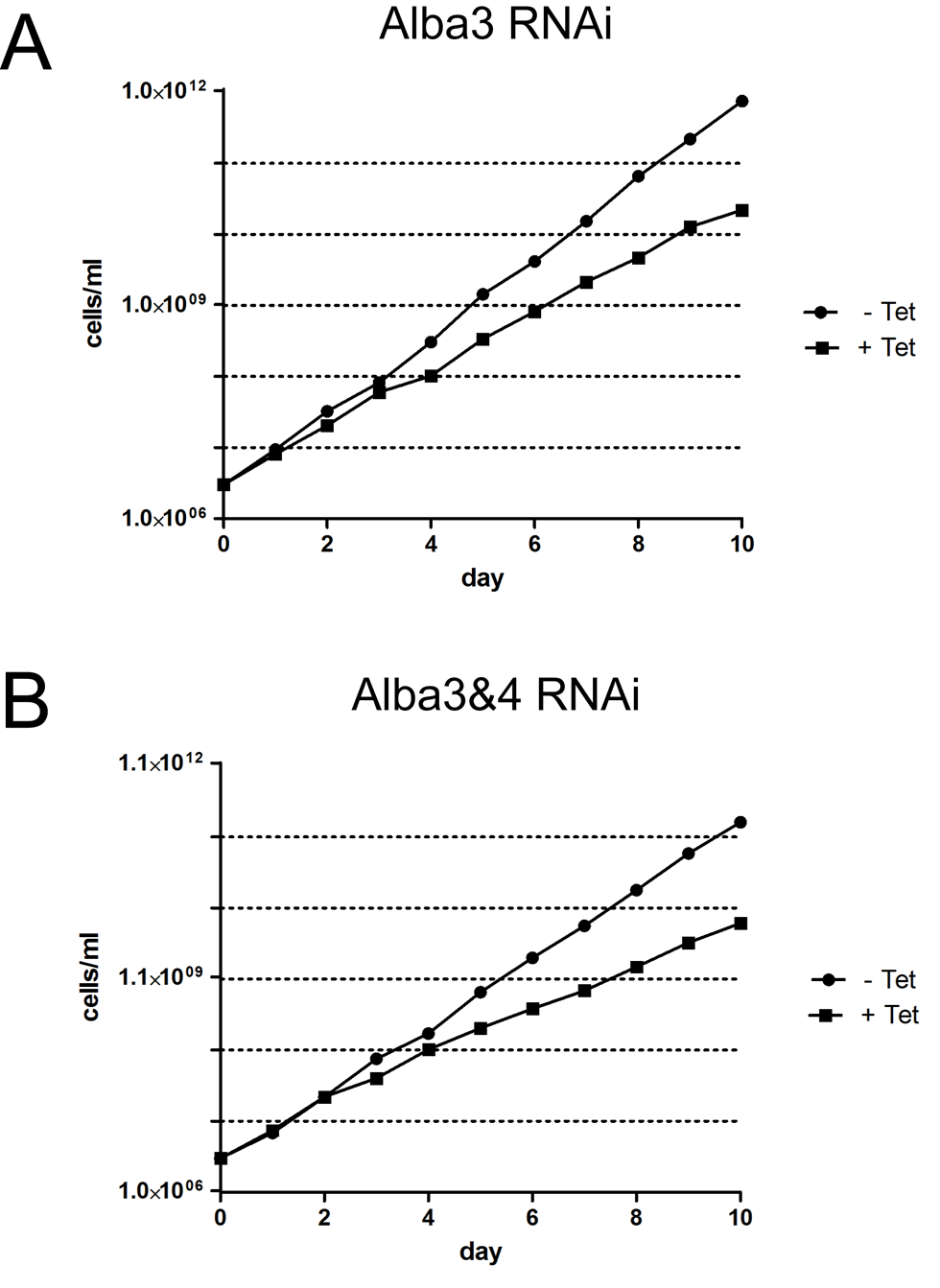

Supplement: Figure S3 — Knockdown of Alba3 or Alba3&4 slows growth. Growth of (A) Alba3 and (B) Alba3&4 RNAi cells in DTM + 15% FBS was monitored for 10 days without induction (- Tet, black circles) or with induction by tetracycline (+ Tet, black squares). Cells were counted and diluted to 3×106 ml−1 daily. Graphs show the cumulative cell number per ml medium. (TIF) [file pone.0022463.s003.tif]

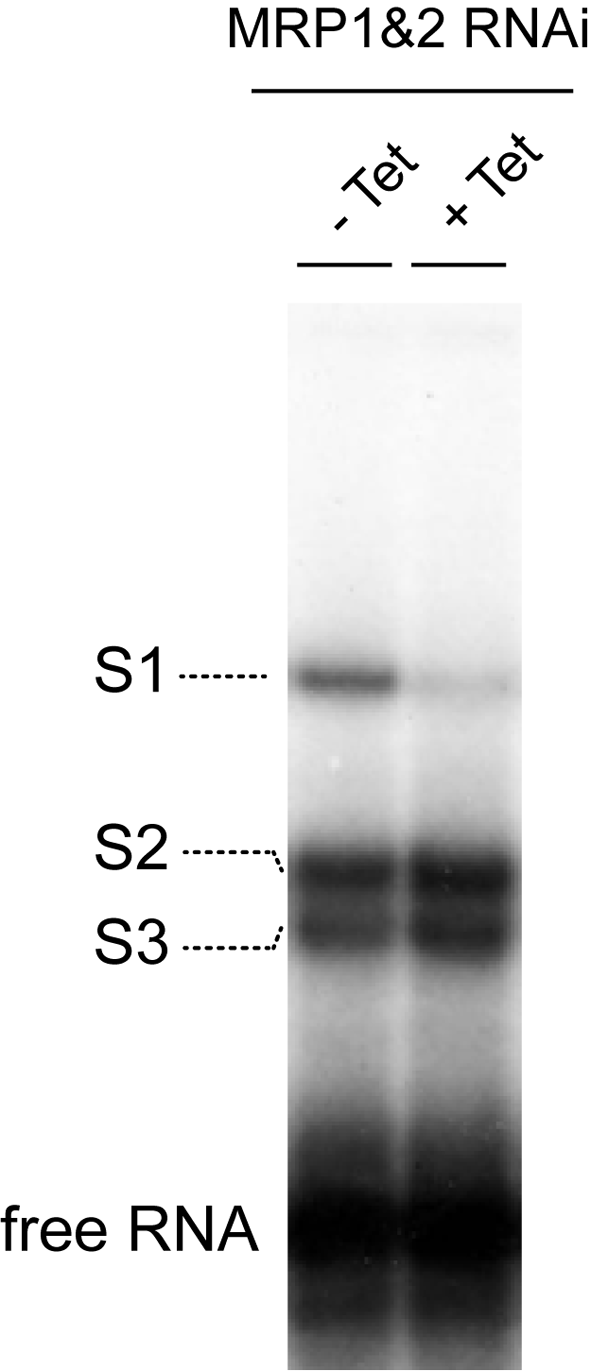

Supplement: Figure S4 — MRP1 and MRP2 interact with GPEETLII RNA in vitro . Protein extracts from uninduced (- Tet) and induced (+ Tet) cultures of MRP1&2 RNAi were incubated in the presence of 32P-GPEETLII RNA followed by separation on 10% native polyacrylamide gels. Ablation of MRP1 and MRP2 led to marked reduction of the S1 band shift compared to the control cells after 3 days of induction. (TIF) [file pone.0022463.s004.tif]

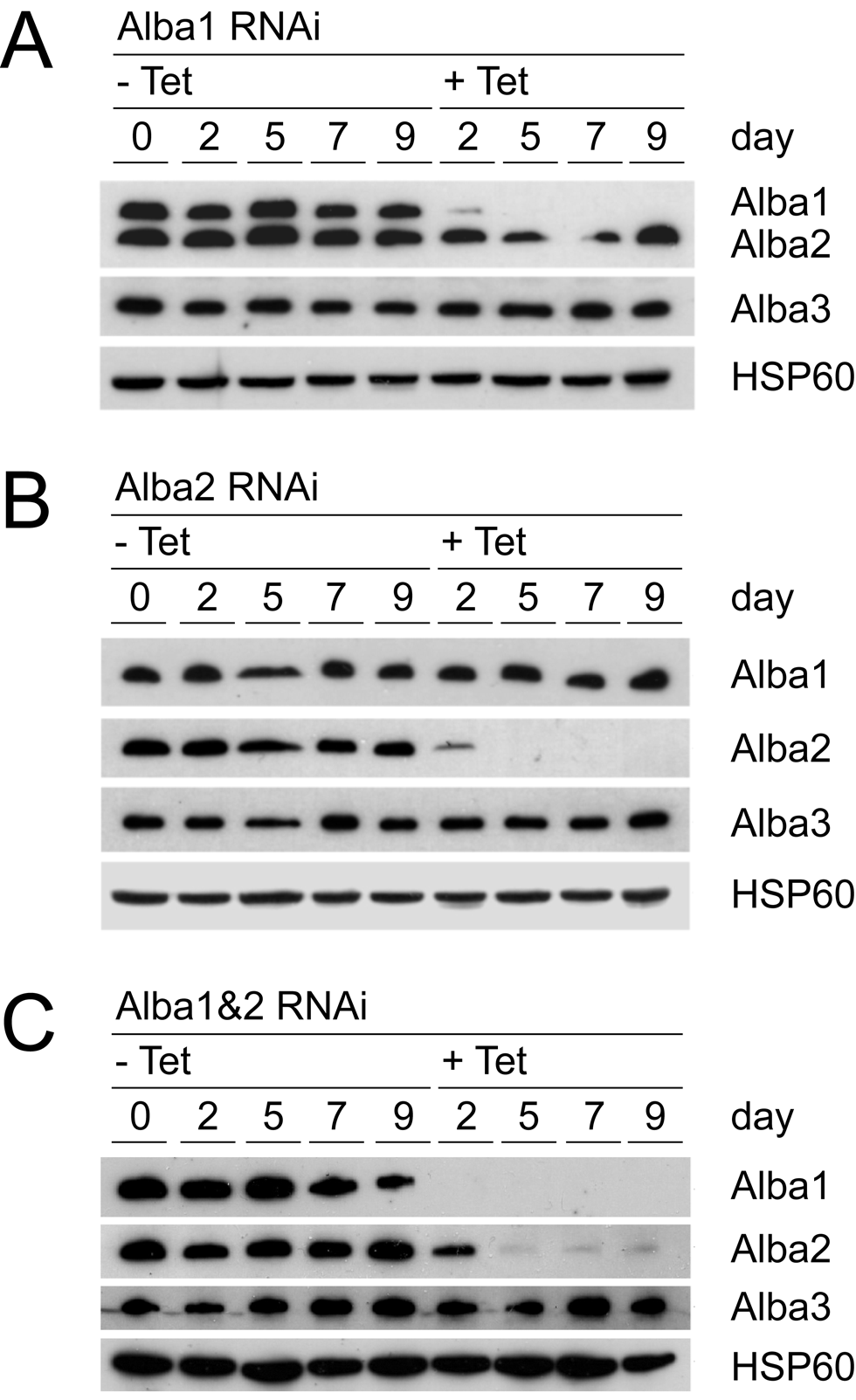

Supplement: Figure S5 — Knockdown of Alba1 and Alba2 does not affect Alba3 protein levels. Western blots using antibodies specific for Alba1, Alba2 and Alba3 were performed with protein samples from uninduced (- Tet) and induced (+ Tet) cells. (A) Alba1, (B) Alba2, (C) Alba1&2 RNAi. HSP60 served as loading control. (TIF) [file pone.0022463.s005.tif]

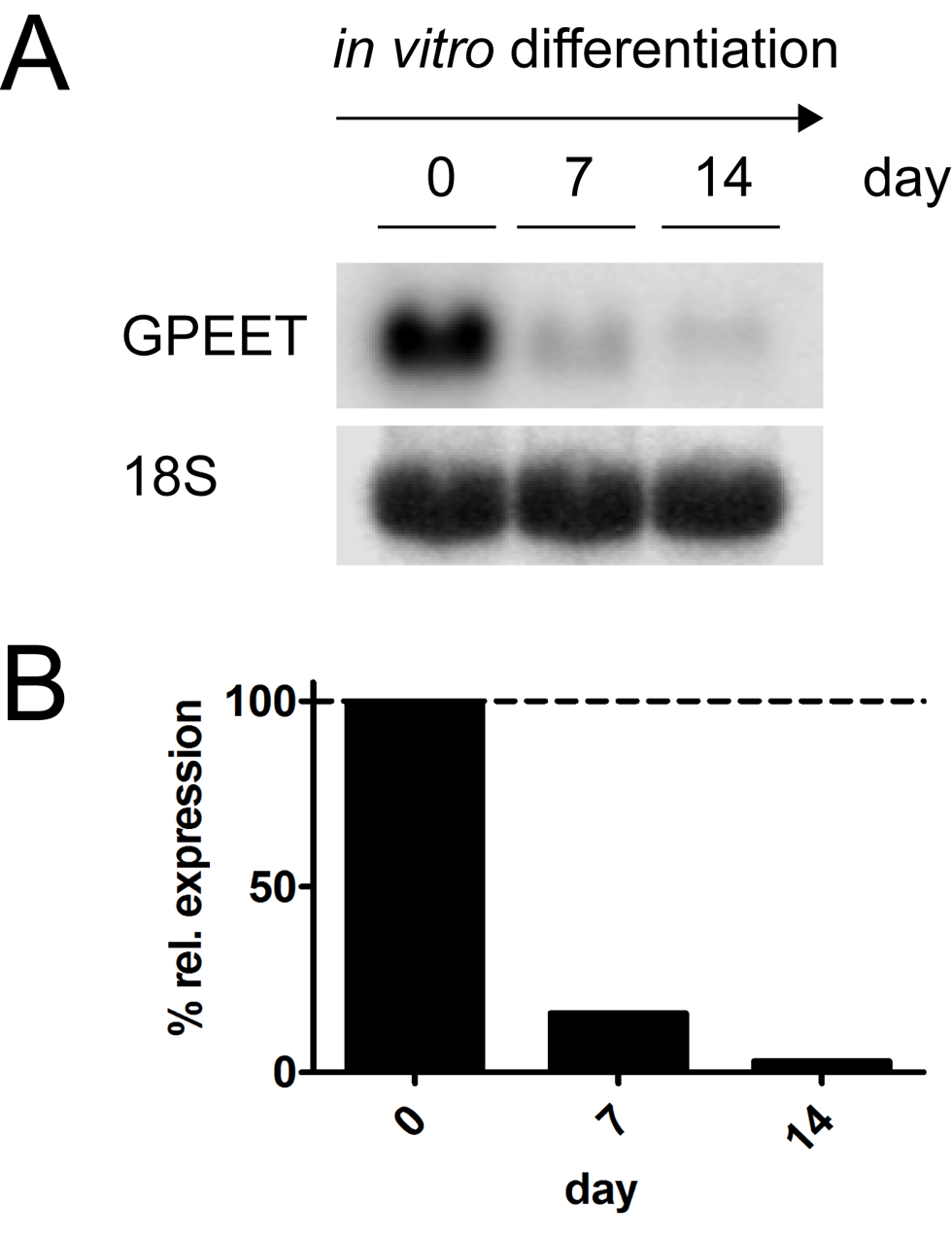

Supplement: Figure S6 — Northern blot analysis of GPEET mRNA during differentiation of early procyclic forms to late procyclic forms. (A) Total RNA was isolated at the times indicated after removal of glycerol from the medium. Hybridisation conditions and probes for GPEET and 18S were as for Figure 8. (B) Quantification of signals after normalisation to 18S rRNA. (TIF) [file pone.0022463.s006.tif]

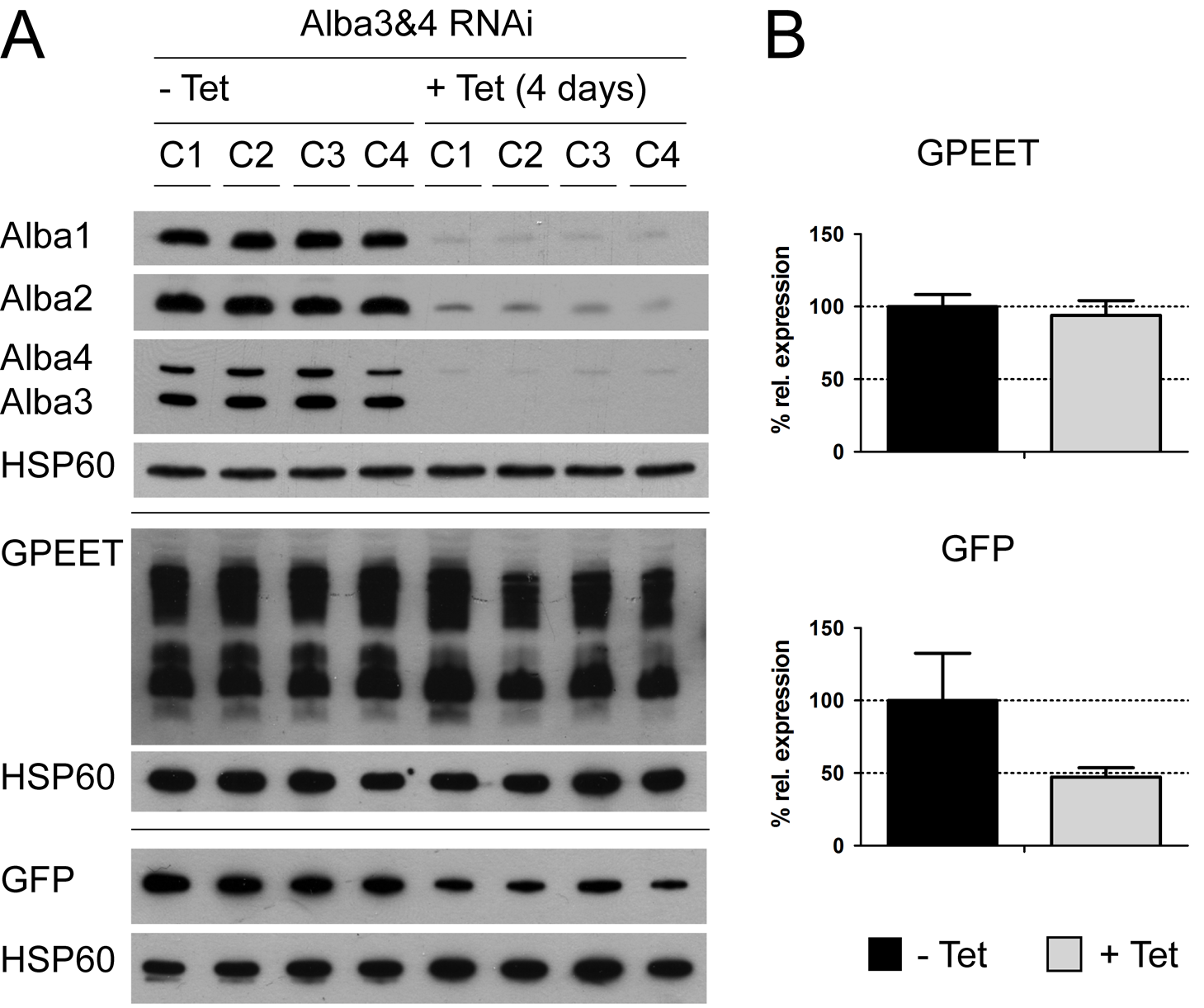

Supplement: Figure S7 — Comparison and quantification of GPEET and GFP protein levels in uninduced and induced cultures of Alba3&4 RNAi cells. (A) Western blots were performed with the appropriate antibodies (see Materials & Methods). (B) GPEET and GFP levels were normalised against HSP60. (TIF) [file pone.0022463.s007.tif]

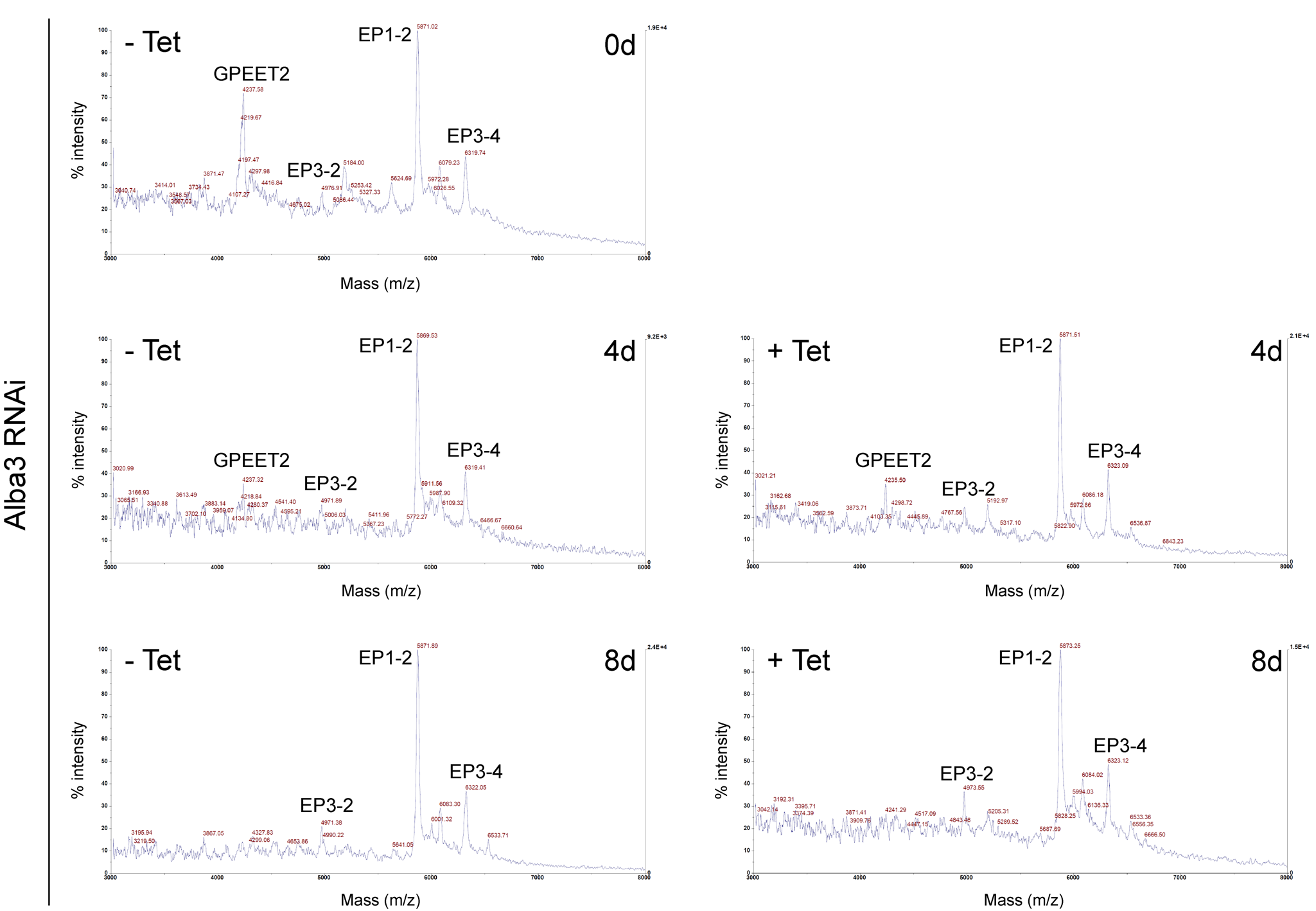

Supplement: Figure S8 — Analysis of procyclin isoforms after knockdown of Alba3. Procyclins were analysed in uninduced (- Tet) and tetracycline-induced (+ Tet) Alba3 RNAi cells over a period of 8 days. Negative ion MALDI-TOF spectra show the major procyclin isoforms. GPEET expression varies in culture, but is not affected by knockdown of Alba3. C1 -C4 denote independent cultures. (TIF) [file pone.0022463.s008.tif]

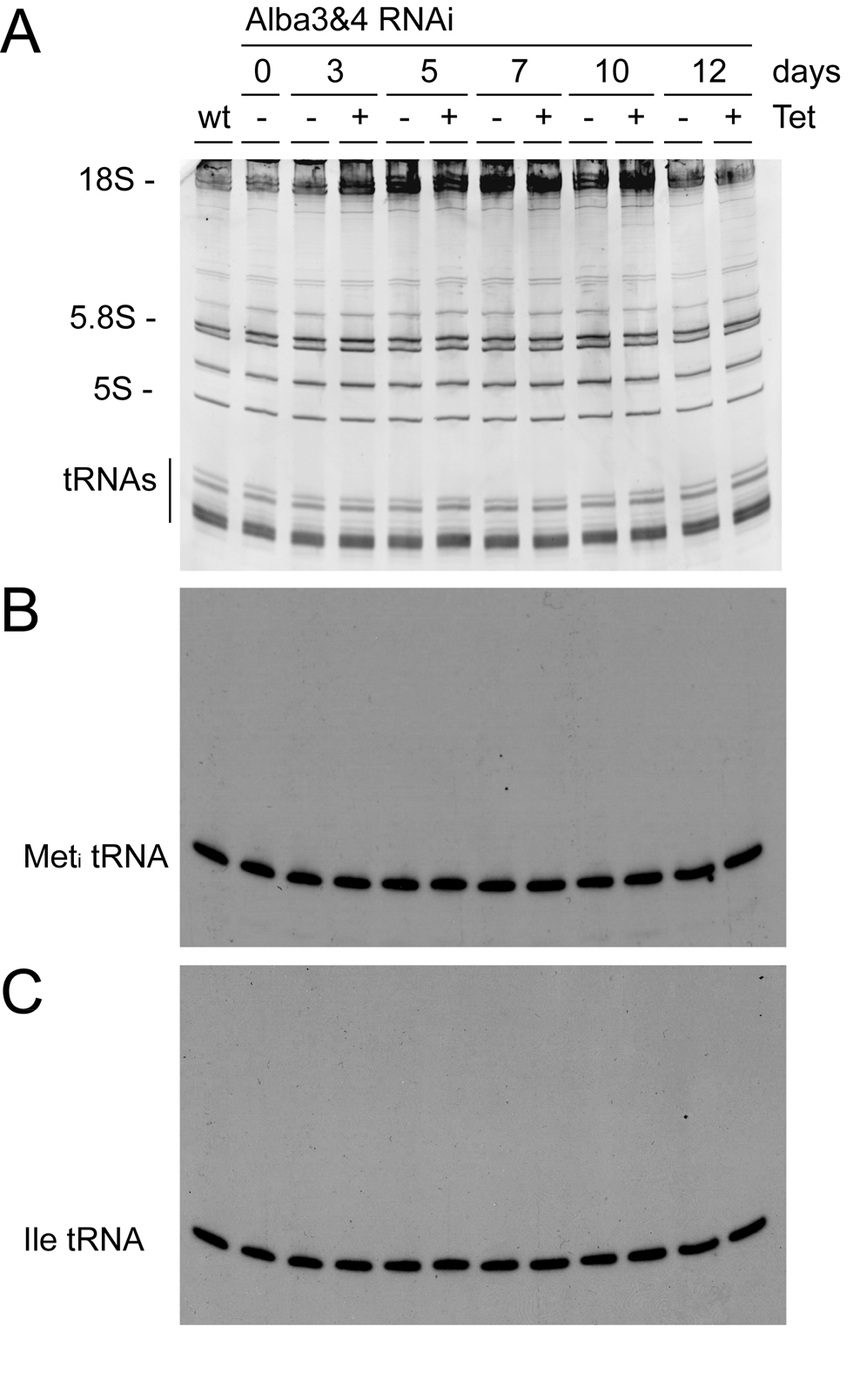

Supplement: Figure S9 — Downregulation of Alba proteins does not lead to accumulation of tRNA precursors. (A) RNA from wild type AnTat1.1 (wt) and Alba3&4 RNAi cells cultured in the presence (+Tet) or absence (-Tet) of tetracycline was separated on polyacrylamide gels and stained with ethidium bromide. Representative RNA species are indicated on the left. Northern blots were performed with probes recognizing methionyl initiator (Meti) tRNA (B) and isoleucine (Ile) tRNA (C). (TIF) [file pone.0022463.s009.tif]

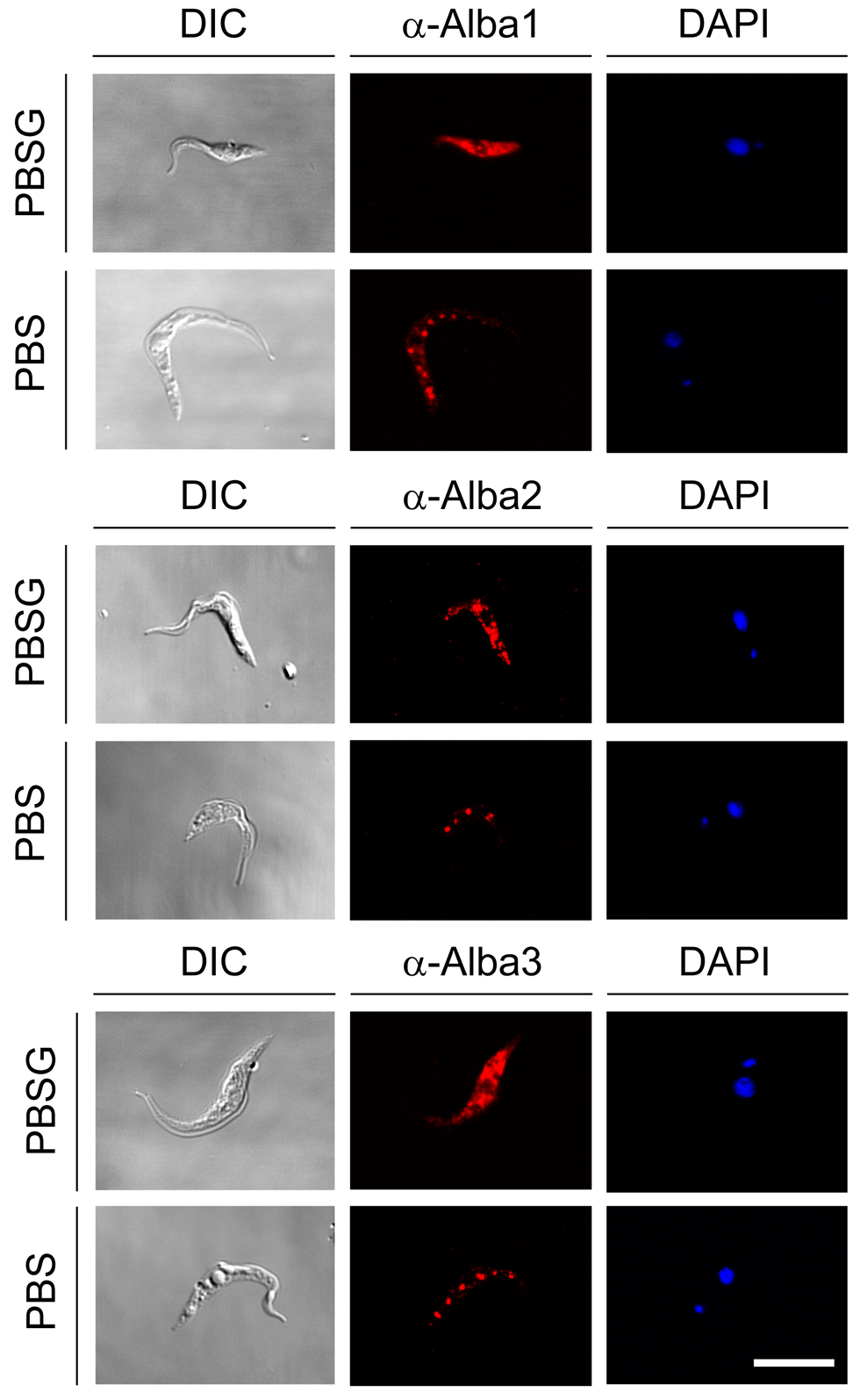

Supplement: Figure S10 — Immunofluorescence analysis of Alba1, Alba2 and Alba3. Alba1, Alba2 and Alba3 were detected with specific antibodies as indicated. Cells were either incubated for 3 h in PBS or in PBS + glucose (PBSG). The scale bar represents 10 mm. DIC: Differential interference contrast. DAPI: 4,6-diamidino-2-phenylindole. Samples were analysed with a Leica DM IRE2 inverted microscope connected to a Leica True Confocal Scanner. (TIF) [file pone.0022463.s010.tif]

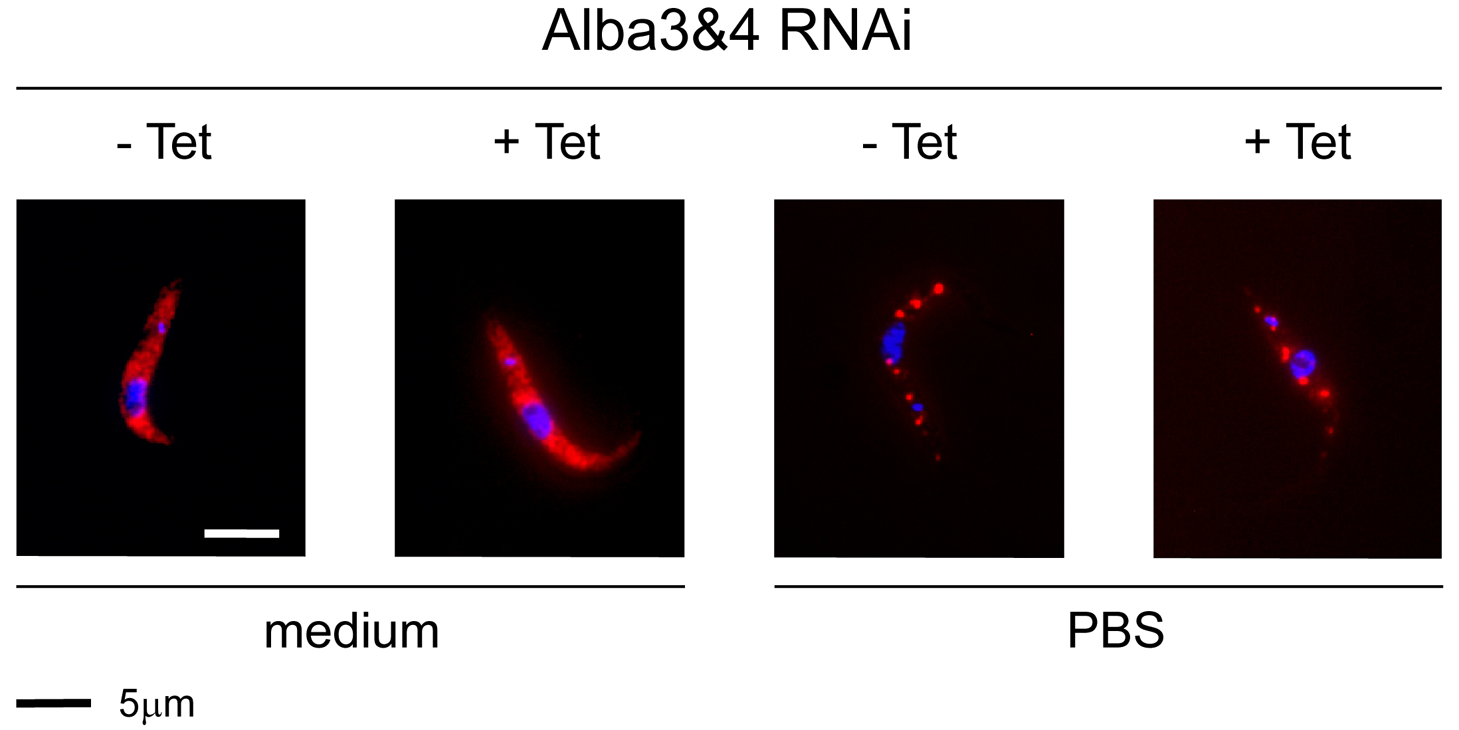

Supplement: Figure S11 — Alba proteins are dispensable for stress granule (SG) formation. Alba3&4 RNAi cells were cultured for 3 days in the presence (+ Tet) or absence (- Tet) of tetracycline. Knockdown of Alba 3&4 also causes depletion of Alba 1 and Alba2. Parasites were stressed by incubation in PBS for 3 h or kept in normal medium. Poly(A) RNA was detected by fluorescence in situ hybridisation using a Cy3-labeled oligod(T)30 probe. Nuclei and kinetoplasts were visualized by DAPI staining. (TIF) [file pone.0022463.s011.tif]

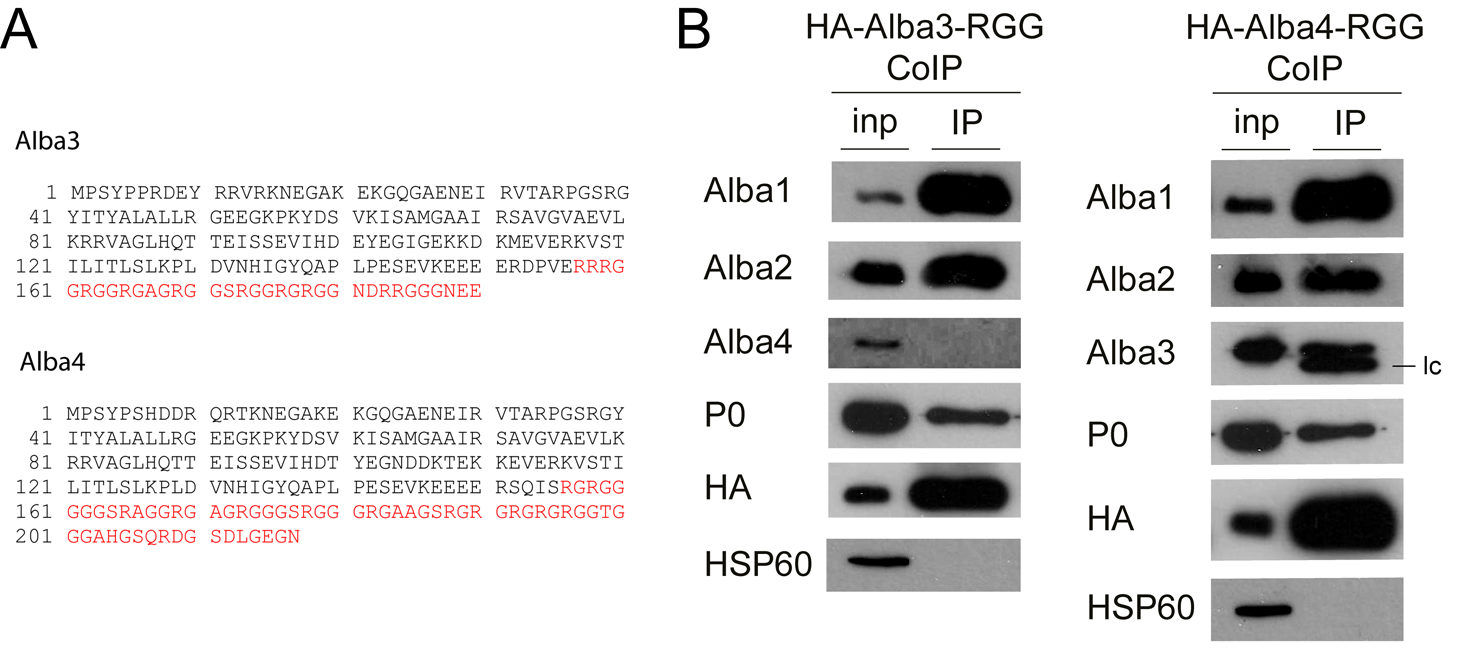

Supplement: Figure S12 — RGG repeats of Alba3 and Alb4 are dispensable for interactions with Alba proteins and P0. (A) HA-tagged Alba3 and Alba4 lacking the carboxy-terminal RGG repeats (shown in red) were ectopically expressed. (B) Protein samples corresponding to the input (inp) and precipitated (IP) material were analyzed by immunoblotting with the antibodies indicated. Anti-HA served as a positive control for the pulldown and HSP60 as a negative control. lc indicates signals from the light chain of the anti-HA antibody used for the pulldown. (TIF) [file pone.0022463.s012.tif]
